# Comparisons of the Effects of Elevated Vapor Pressure Deficit on Gene Expression in Leaves among Two Fast-Wilting and a Slow-Wilting Soybean

**DOI:** 10.1371/journal.pone.0139134

**Published:** 2015-10-01

**Authors:** Mura Jyostna Devi, Thomas R Sinclair, Earl Taliercio

**Affiliations:** 1 Department of Crop Science, North Carolina State University, Raleigh, North Carolina, United States of America; 2 Soybean and Nitrogen Fixation Unit, USDA-ARS, Raleigh, North Carolina, United States of America; Estación Experimental del Zaidín (CSIC), SPAIN

## Abstract

Limiting the transpiration rate (TR) of a plant under high vapor pressure deficit (VPD) has the potential to improve crop yield under drought conditions. The effects of elevated VPD on the expression of genes in the leaves of three soybean accessions, Plant Introduction (PI) 416937, PI 471938 and Hutcheson (PI 518664) were investigated because these accessions have contrasting responses to VPD changes. Hutcheson, a fast-wilting soybean, and PI 471938, a slow-wilting soybean, respond to increased VPD with a linear increase in TR. TR of the slow-wilting PI 416937 is limited when VPD increases to greater than about 2 kPa. The objective of this study was to identify the response of the transcriptome of these accessions to elevated VPD under well-watered conditions and identify responses that are unique to the slow-wilting accessions. Gene expression analysis in leaves of genotypes PI 471938 and Hutcheson showed that 22 and 1 genes, respectively, were differentially expressed under high VPD. In contrast, there were 944 genes differentially expressed in PI 416937 with the same increase in VPD. The increased alteration of the transcriptome of PI 416937 in response to elevated VPD clearly distinguished it from the other slow-wilting PI 471938 and the fast-wilting Hutcheson. The inventory and analysis of differentially expressed genes in PI 416937 in response to VPD is a foundation for further investigation to extend the current understanding of plant hydraulic conductivity in drought environments.

## Introduction

Drought is a major abiotic stress that limits plant growth and drastically reduces crop productivity. Mechanisms that impart tolerance to drought in plants include conservation of soil water through reduced water loss, reduced radiation absorption, reduced leaf area, lower lethal relative water content, deep rooting and lower stomatal conductance [1,2].

A key factor in the genetic improvement of soybean yield under drought conditions has been the discovery of the slow-wilting trait [3]. Two exotic soybean genotypes, PI 416937 and PI 471938, have been identified as “slow to wilt” when subjected to drought in the field [3]. In contrast to the two slow-wilting genotypes, nearly all other soybean genotypes exhibit wilting in the field 4 to 5 days prior to the two slow-wilting lines as drought conditions develop [3]. When these two slow-wilting genotypes were exposed to various levels of VPD, PI 471938 showed continued increases in TR as VPD increased, similar to a fast-wilting control. This increasing transpiration rate with increasing VPD is the commonly observed response [4,5]. However, PI 416937 was found to have the unusual ability to restrict its TR when the surrounding air was dry or at a high VPD of above 2 kPa [5,6,7]. It was discovered that this limited TR under high VPD was associated with low hydraulic conductance in the leaves under high atmospheric VPD [6].

Limited TR expressed under high VPD by the slow-wilting phenotype of PI 416937 would be especially desirable in dry (high VPD) environments where water deficits commonly develop in the latter part of the growing season. In these environments, a restricted TR during the middle of the day, induced by a high VPD, would result in water conservation early in the growing season, improved water use efficiency and, thus, increased yield [8]. Computer simulations modeling the limited-TR trait under high VPD indicated yield increases for soybean in most US environments [8].

To take advantage of the slow-wilting trait in plant improvement programs, it is important to understand the physiological and molecular responses of plants to high VPD. Previous work has evaluated the effects of treatments meant to simulate drought that results in wilting on the transcriptome of PI416937 [9]. The purpose of this study was to compare the responses of the transcriptomes of PI 416937, PI 471938 and Hutcheson to high VPD in a well-watered environment to gain insight into the responses of the slow- and fast-wilting genotypes to changes in VPD. Hence, the inclusion of these three genotypes in this study represented combinations of fast-wilting, insensitive to VPD (Hutcheson), slow-wilting, insensitive to VPD (PI 471938), and slow-wilting sensitive to VPD (PI 416937) in terms of TR in an environment that is not water-limited.

## Methods

Two experiments were conducted to document TR responses of Hutcheson, PI 471938 and PI 416937 to VPD. The samples for transcriptome profiling were collected in the second experiment.

### Transpiration response to VPD

Transpiration response to VPD was measured by adapting an approach used previously [5]. S1 Fig shows the experimental set-up. Plants were grown in pots made of polyvinyl chloride pipe (100-mm diam., 180-mm tall) in a greenhouse (Phytotron, North Carolina State University, Raleigh, NC, 35´46°N; 78´39°W). A small hole was drilled in the bottom of each pot, which allowed drainage of any excess water. The top of the pot was fitted with a toilet flange to allow easy attachment of a VPD chamber during measurement.

The pots were filled with Gardenplus topsoil (#92432, Lowes Inc., North Wilkesboro, NC), containing 14–6.1–10 N-P-K fertilizer. Three seeds were sown per pot and inoculated with *Bradyrhizobium* (Nitragin, Inc., Brookfield, WI). Each pot was thinned to one plant after a week of germination. Plants were well-watered, and the air temperature of the greenhouse was regulated at 30°C day/26°C night.

VPD treatments were begun when the plants developed approximately four fully developed leaves. The evening before TR measurements, each pot was moved into a walk-in growth chamber (30°C) and watered to dripping. Aluminum foil was placed over the soil and sealed around the stem of the plant to eliminate evaporation from the soil surface. The photon flux density in the growth chamber was about 550 μmol m^-2^ s^-1^. In the first and second experiments, four replications for each genotype were included to study transpiration response to VPD; each replicate represented one plant. Experiment 1 began on 28 March 2012 and Experiment 2 on 17 June 2012.

Plants were prepared for measurement by enclosing each in a 340-mm diameter, 21-L transparent food container (Cambro Manufacturing, Huntington Beach, CA), which was attached to the toilet flange of the pot. The connection between the humidity test chamber and the toilet flange was not sealed to allow air flow through the chamber. Each humidity chamber was fitted with a 12-V, 76-mm-diameter computer box fan (Northern Tool and Equipment, Burnsville, MN) to continuously stir the air inside the chamber. A humidity/temperature data logger (Lascar Electronics, Erie, PA) was mounted through the side wall of each container to record the chamber environment.

Various humidity levels were achieved in the chambers by flowing air at different volume rates and sources to balance the humidifying effect of the transpiring leaves. For each block of plants, the plants were subjected to three VPD levels: low (0-1kPa), medium (1.5–2.5 kPa), and high (2.5–4 kPa). Plants were allowed to stabilize for 30 min after introducing each humidity treatment. The plants were subjected to one hour at each VPD level in Experiment 1 and for four hours in each VPD level in Experiment 2. The longer exposure to each VPD level in Experiment 2 was done to allow full gene response. In Experiment 1, transpiration rates were calculated as the difference in weights taken every hour at each VPD level. In Experiment 2, transpiration rates were calculated as the difference in the weights taken for four hours. The VPD during each treatment in each chamber was calculated on the basis of temperature and relative humidity measured during each observation period. After completing measurements in the second experiment, second leaves were collected for RNA isolation from three replicates and stored at -80°C. The leaves remaining were separated from the stem and leaf area was measured using a leaf area meter (LI-1300, Licor, Lincoln, NE). TR was expressed as water-loss rate divided by plant leaf area.

All of the TR data for each genotype was first examined by attempting to fit the data to a two-segment linear regression using GraphPad Prism 5.0 (GraphPad Software Inc., San Diego, CA). The outputs of a successful regression that fit to the two-segment model were the coefficients defining two intersecting linear regressions at the break point (BP):

If VPD < BP, TR = slope 1(VPD) + intercept 1

If VPD ≥ BP, TR = slope 2 (VPD) + intercept 2

The slopes of the two linear regressions (Slope1 and 2) were statistically compared to determine if they differed significantly (*p* < 0.05). If the slopes differed, the two-segment linear regression was retained. When the slopes were not significantly different, a single linear regression was applied to all the data.

### Sample collection and RNA isolation

Samples for the RNA isolation were collected from Experiment 2. Leaf samples were collected from each genotype in at least three replications each after exposing the plants for four hours to low and high VPD levels. Total RNA was extracted from the leaf tissues using Qiagen Rneasy plant kit according to the manufacturer's instructions. After DNaseI treatment (Ambion), RNA was quantified and quality confirmed using Bioanalyzer 2100 (Agilent, Inc., Palo Alto, CA, USA). TruSeq (Illumina) libraries were constructed using this RNA.

### Transcriptome profiling and data analysis

TruSeq RNA libraries from two replicates of each treatment for each genotype were sequenced by HiSeq (Illumina). The number of 125 bp sequence reads recovered and aligned to the reference genome using TopHat ranged between 12 million and 18 million with more than 76% of sequences aligning to the reference soybean genome. Details were shown in S1 Table. The annotation of the reference genome was assigned to the aligned sequences. Transcripts that varied 2 fold or greater and were statistically significant across reps (q<0.05) were considered differentially regulated by VPD.

Subsequently, 944 up/down-regulated genes in PI 416937 due to high VPD were used for functional annotation based on Gene Ontology (GO) categories using singular enrichment analysis (SEA) by AgriGo (http://bioinfo.cau.edu.cn/agriGO/FAQ.php). To assess the significance of over-represented GO terms in the list of the significantly regulated genes against all regulated genes in PI 416937, Fisher's Test (p < 0.01) and Yekutieli method (FDR < 0.05) were used. The visualization of profiling data of 944 differentially expressed genes in the context of various pathways overview was performed with MapMan along with their Log2-fold change values (www.mapman.com). The Gmax_109_peptidemapping file was used to map the genes responsive to high VPD onto various pathways using the image annotator module of the MapMan application.

### Real time quantitative PCR

Genes were selected to validate the differential expression of genes in PI 416937 and PI 471938. Gene XM_003528209 [UKN 2 (Gene of unknown function)] from soybean refseq at NCBI was used to normalize all values in the QRT-PCR assays. Primers for QRT-PCR were designed using Primer3 software and for normalizer from the literature [10]. Primer sequences and efficiencies are available in Table 1.

**Table 1:** List of genes used in QRT PCR for the validation of transcriptome data, amplification efficiency and their primer sequence.

Gene	Amplification efficiency	Forward	Reverse
Glyma16g08340	2.07	ATGTATTCTCAAGACCCAAATG	GAGTTGAGCTTTGTTCAGCAC
Glyma18g52480	1.84	AGTGTGGCAGCGTGATCGA	TGGTAGCAGCCTGGTTCTTG
Glyma08g05500	1.82	AGCGAAGAGTACAGGAAAGTG	CCTTCTTCAGGTACCCCTTC
Glyma03g37940	1.90	GATTTCCCTCTATCGTCACAC	GTTCGGAGTCGCAGGTTGC
Glyma13g36870	2.02	GATTTGGCGTGTTTGAACTTTG	GCTGATAATTGTTGTTGCTACC
Glyma13g35560	2.05	TGAGGATGTGGCGGGTACC	TCCATCACCTATTGACCCCAAT
Glyma17g17210	1.90	CGATGCCACCAACACCAGTG	TGGAGAAGCTGCTGAGGCTT
Glyma0892s00200.1	2.00	TAAGGGACACACCAAGCGAA	CAACGTGTCCATCCTTATCC
Glyma05g35050	1.80	CAAAGAATCATCTTCTTCAGC	GTTGAATGCTGAGAAACACC
Glyma10g33060	2.00	AACCGAGGAGCACTCCGG	CCGCCGTACGCCGCG
XM_003528209.2	2.02	GCC TCT GGA TAC CTG CTC AAG	ACC TCC TCC TCA AAC TCC TCT G

QRT-PCR reactions based on SYBR Green fluorescence were performed using SYBR GreeniTaq Universal probes one-step kit, 500x20 μl reactions (# 172–5141, Bio-Rad, Hercules, CA, USA) on a Bio-Rad CFX Real-Time PCR Detection System (Bio-Rad) following the manufacturer's instructions. One microliter of synthesized cDNA (diluted 1:10) was used as template. Primer efficiency was determined as explained by Pfaffl [11]. The amplification reactions consisted of a 2-min denaturing step at 95°C, followed by 40 cycles at 95°C for 10 s, 30 s at the primer binding temperature of 56°C and product extension at 70°C for 30 s. After 40 cycles, the program ended with the melting curve program. Analysis of the melt curve was consistent with a single amplicon. Three replicate reactions per sample were used to ensure statistical significance. Expression levels for all candidate genes were computed based on the stable expression level of the reference gene using the method described by Pfaffl [11].

### Availability of Supporting Data

Sequence data was deposited in GEO: GSE49097.

## Results and Discussion

### Transpiration response to VPD

Two experiments were conducted to confirm the TR response to VPD in all three soybean genotypes and to provide material for transcriptome analyses. The temperature range in Experiment 1 was 26°C to 34°C, and in Experiment 2, it was between 27°C and 34°C (S2 Table). In all the experiments, desired VPD levels were obtained by controlling the relative humidity levels between 15 to 86%. By pumping moist or dry air into the plant-test chambers, minimum and maximum VPDs obtained were 0.77 and 3.58 kPa for all genotypes in Experiment 1. In Experiment 2, the low VPD obtained in the plant-test chambers was between 0.7 and 1.2 kPa, and the high VPD range was between 3 and 3.5 kPa. The leaf samples for molecular studies were collected from plants exposed to these different VPDs.

**Fig 1:**
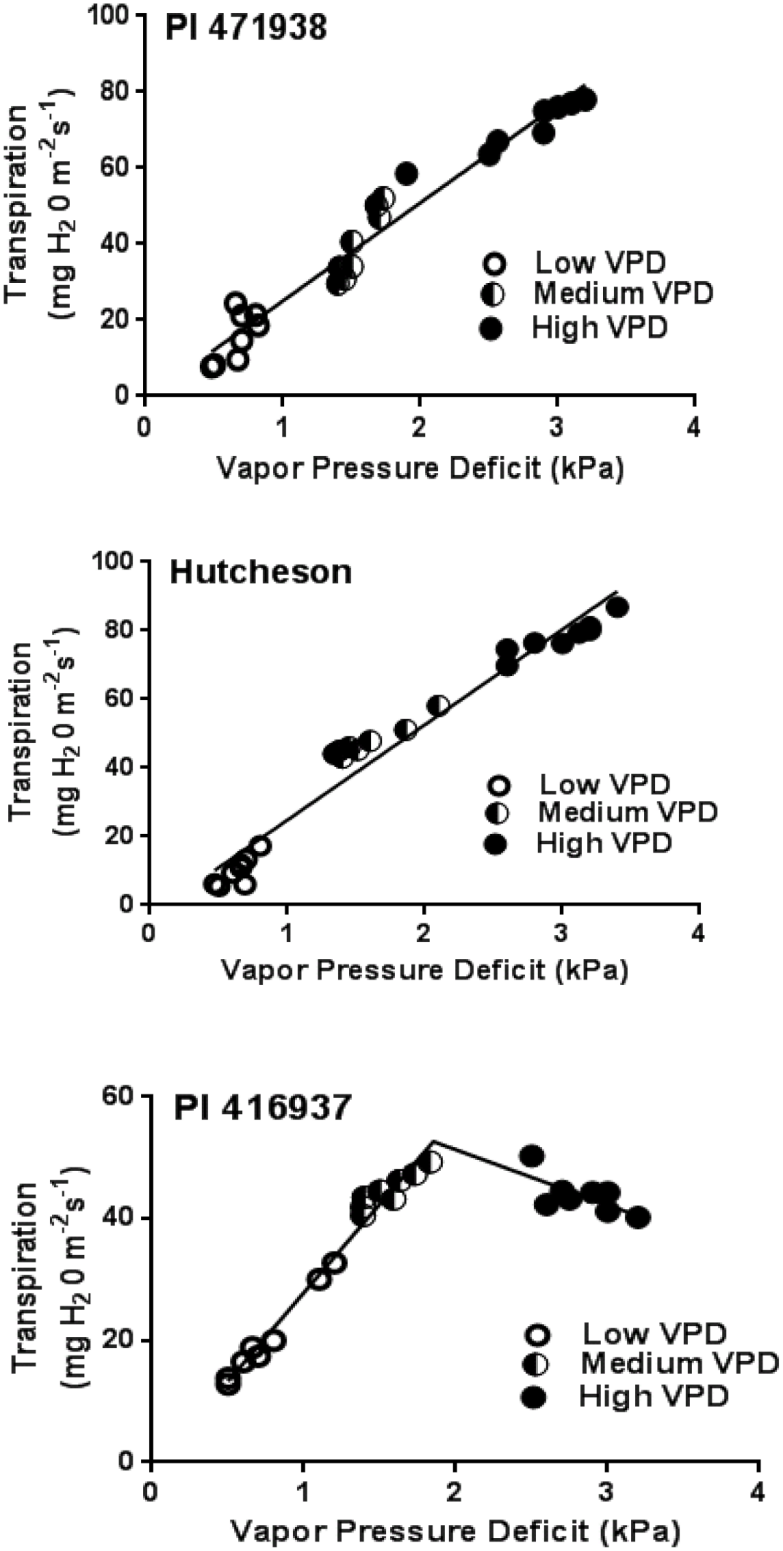
Transpiration rate (TR) response of (a) Hutcheson (b) PI 471938 and (c) PI 416938 to low, medium and high vapor pressure deficit (VPD) conditions from experiments 1 and 2 (4 replicates in each experiment at each VPD level). X-axis represents VPD levels, and the Y-axis represents corresponding transpiration rates. Closed, half closed and open circles are TR values under low, medium and high VPD conditions, respectively. Data points are the individual plant TRs exposed to various VPDs. The regressions in (a) and (b) are single linear relationships, and the regression in (c) is a two-segmental nonlinear relationship. More details are shown in S2 Table.

Among the three genotypes, only PI 416937 showed the limited or altered TR response to VPD. The response of PI 416937 was best described by a two-segment regression model with a VPD break point at about 2 kPa VPD (Fig 1). By contrast, TR of the genotypes PI 471938 and Hutcheson displayed the typical linear response of soybean over the entire range of VPD conditions, as has been reported previously [5,7].

### Transcriptome profiling

**Fig 2:**
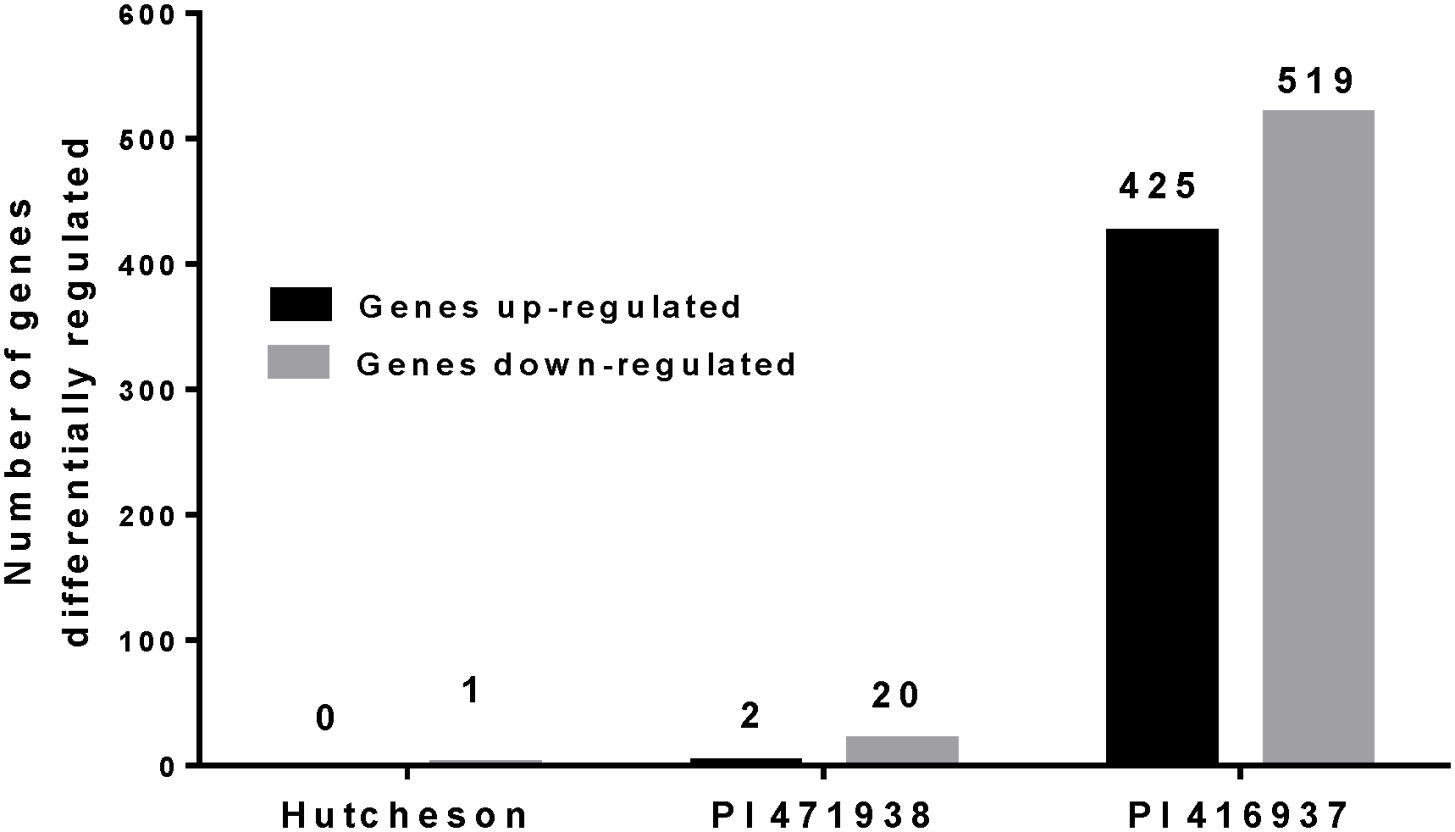
Number of genes differentially expressed in leaves of Hutcheson, PI 471938 and PI 416937 exposed to high VPD. Black bars represent number of genes up-regulated in each genotype, and grey bars correspond to number of down-regulated genes.

RNA was isolated from the second leaf from the apical meristem from plants of all three genotypes that had been exposed to a low VPD environment (0.7 to 1.2 kPa) or challenged by a VPD above 2 kPa (3 to 3.5 kPa). We compared gene expression within the genotypes and between the treatments to understand the differences in the TR responses of the soybean genotypes to high VPD conditions. These comparisons identified the genes differentially regulated under elevated VPD conditions in the three genotypes. A total of 39,068 genes were expressed in leaves of soybean plants exposed to low or high VPD environments. The high total number of expressed genes probably reflects the fact that genes expressed in any genotype with low levels of expression in both treatments were included. To identify the gene expression pattern between the treatments, the numbers of up-regulated and down-regulated genes were calculated for high VPD samples in comparison to low VPD samples. The fast-wilting Hutcheson and the slow-wilting PI 471938 transcriptomes were fairly unresponsive to an increase in VPD with significantly (q<0.05) altered expression of only 1 and 22 transcripts, respectively (Fig 2 and Table 2). The transcriptome of PI 416937 was markedly more responsive to a comparable increase in VPD with a significant change in expression of over 900 transcripts (p<0.05) (Fig 2 and S3 Table).

**Table 2:** Differentially expressed genes in PI 471938 and their functional annotations.

Phytozome transcript Name	Tentative annotations	Log2-Fold Change
Glyma01g31300.1	ATFER1; ferric iron binding / iron ion binding	-5.29974
Glyma01g44930.1	MSS1; carbohydrate transmembrane transporter/ hexose:hydrogen symporter/ high-affinity hydrogen:glucose symporter/ sugar:hydrogen symporter	-4.01891
Glyma02g03230.1	matrixin family protein	-4.58977
Glyma04g05720.1	HSP17.6II (17.6 KDA CLASS II HEAT SHOCK PROTEIN)	-5.27341
Glyma04g40600.1	DMR6 (DOWNY MILDEW RESISTANT 6); oxidoreductase/ oxidoreductase, acting on paired donors, with incorporation or reduction of molecular oxygen, 2-oxoglutarate as one donor, and incorporation of one atom each of oxygen into both donors	-5.67626
Glyma06g45520.1	MYB14 (MYB DOMAIN PROTEIN 14); DNA binding / transcription factor	-5.60384
Glyma07g19060.1	ATFER1; ferric iron binding / iron ion binding	-4.63384
Glyma0892s00200.1	ankyrin repeat family protein	-4.37683
Glyma08g02940.1	BIP2; ATP binding	-3.86183
Glyma08g21410.1	acid phosphatase, putative	-4.62776
Glyma09g24410.1	ATHSP90.1 (HEAT SHOCK PROTEIN 90.1); ATP binding / unfolded protein binding	-5.93546
Glyma09g41050.1	WRKY70; transcription factor/ transcription repressor	-3.79662
Glyma10g01300.1	No Annotation	-6.12145
Glyma14g04530.1	L-ascorbate oxidase/ copper ion binding / oxidoreductase	-5.30679
Glyma14g11920.1	WRKY40; transcription factor	-6.23919
Glyma15g06800.1	ATPRB1	-7.13018
Glyma16g29750.1	ATHSP90.1 (HEAT SHOCK PROTEIN 90.1); ATP binding / unfolded protein binding	-5.33024
Glyma18g48310.1	ankyrin repeat family protein	-4.14359
Glyma18g52470.1	HSP70 (heat shock protein 70); ATP binding	-4.32375
Glyma18g52480.1	HSP70 (heat shock protein 70); ATP binding	-5.06438
Glyma19g06890.1	GDSL-motif lipase/hydrolase family protein	5.83204
Glyma19g43940.1	LTL1 (LI-TOLERANT LIPASE 1); carboxylesterase/ hydrolase, acting on ester bonds	4.79419

Differentially expressed genes in PI 471938 when exposed to high VPD compared to low VPD conditions. Tentative annotation and log2 fold change of twenty down-regulated and two up-regulated genes.

**Fig 3:**
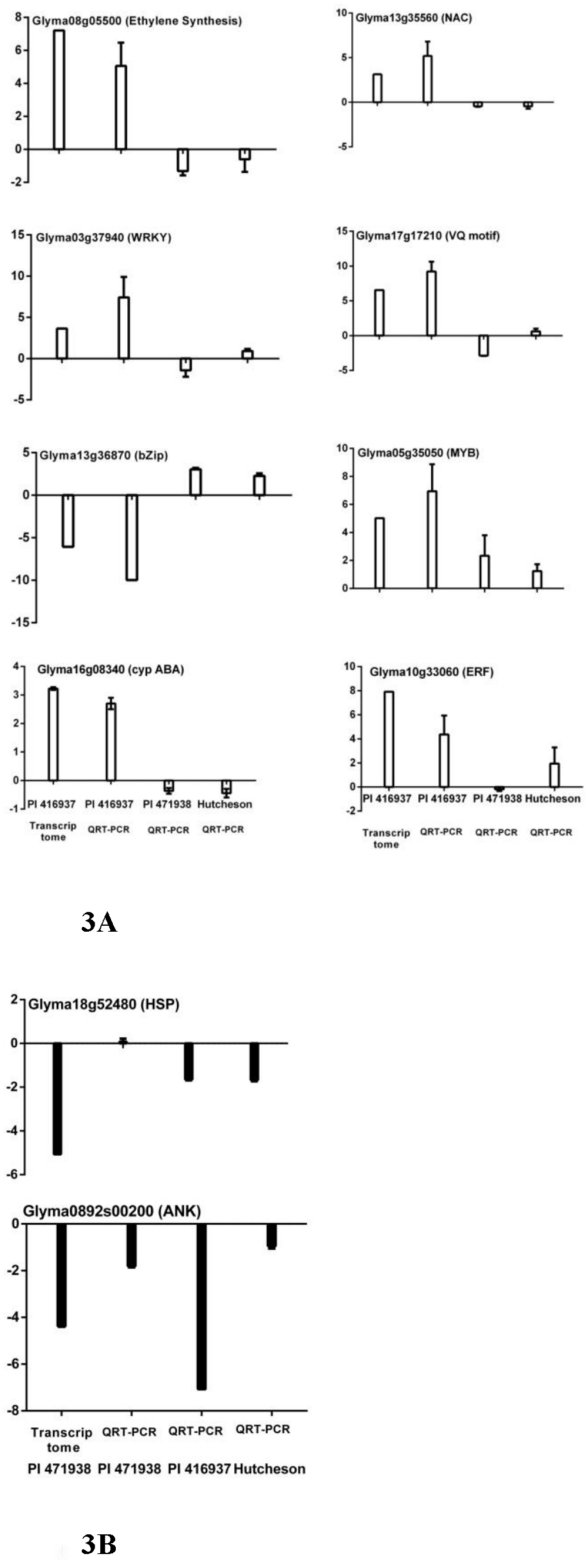
Validation of transcriptome data with QRT-PCR. Genes for the validation were selected from significantly expressed categories of PI 416937 (A) and PI 471938 (B). The X-axis represents soybean genotype, and the Y-axis is log2 fold change of transcript levels at high VPD in comparison to low VPD (q<0.05). The first bars in 3A and 3B represent gene expression data from the transcriptome study in PI 416937 and PI 471938, and remaining bars indicate data from QRT-PCR study. Error bars represent the standard deviation.

The expression of ten genes using QRT-PCR provided validation of the transcriptome profiling data (Fig 3 and S2 Fig). The levels of expression of 8 candidate genes from PI 416937 and 2 candidate genes from PI 471938 were measured by QRT-PCR in replicates treated with high- or low-VPD not used for transcriptome analyses and compared with transcriptome profiling expression (Fig 3). Expression of 6 candidate genes was also validated in one of the replications used for transcriptome profiling (S2 Fig). Even though there was variation in the magnitude of the relative transcript abundance between the profiling data and QRT-PCR data in PI 416937 (Fig 3A), the patterns of accumulation were similar. QRT-PCR confirmed the differential regulation of the ankyrn repeat-containing gene (ANK) at high VPD in comparison with low VPD in PI 471938. However, differential expression of the heat shock protein (HSP) (Glyma18g52480) in PI471938 was not confirmed by QRT-PCR in the third replicate not used for transcriptome profiling (Fig 3B). The differential gene expression of the HSP reported in the transcriptome analysis was apparently a false positive as is expected in large data sets. Additionally, most of the analyses of the transcriptome data rely on the aggregate change of expressions in genes in a pathway and not just an individual gene, making the analysis robust against false positives. The general agreement of the QRT-PCR and transcriptome profiles for 9 out of 10 genes tested confirm the validity of the sequencing data in a third biological replication of the VPD treatments.

Only one gene was significantly differentially regulated in the VPD insensitive cultivar Hutcheson (Fig 2). The location of the gene was Gm08:6264434—‐6265362, and the BlastX search indicated it belonged to the ANK super family. In the other VPD insensitive slow-wilting genotype PI 471938, a total of 22 genes were differentially regulated. Of the 22 differentially regulated genes, 20 were down-regulated, and 2 were up-regulated under elevated VPD (Fig 2, Table 2). Genes differentially regulated in PI 471938 included WRKY, DNA binding transcription factors and ANK (Glyma0892s00200) family proteins.

**Fig 4:**
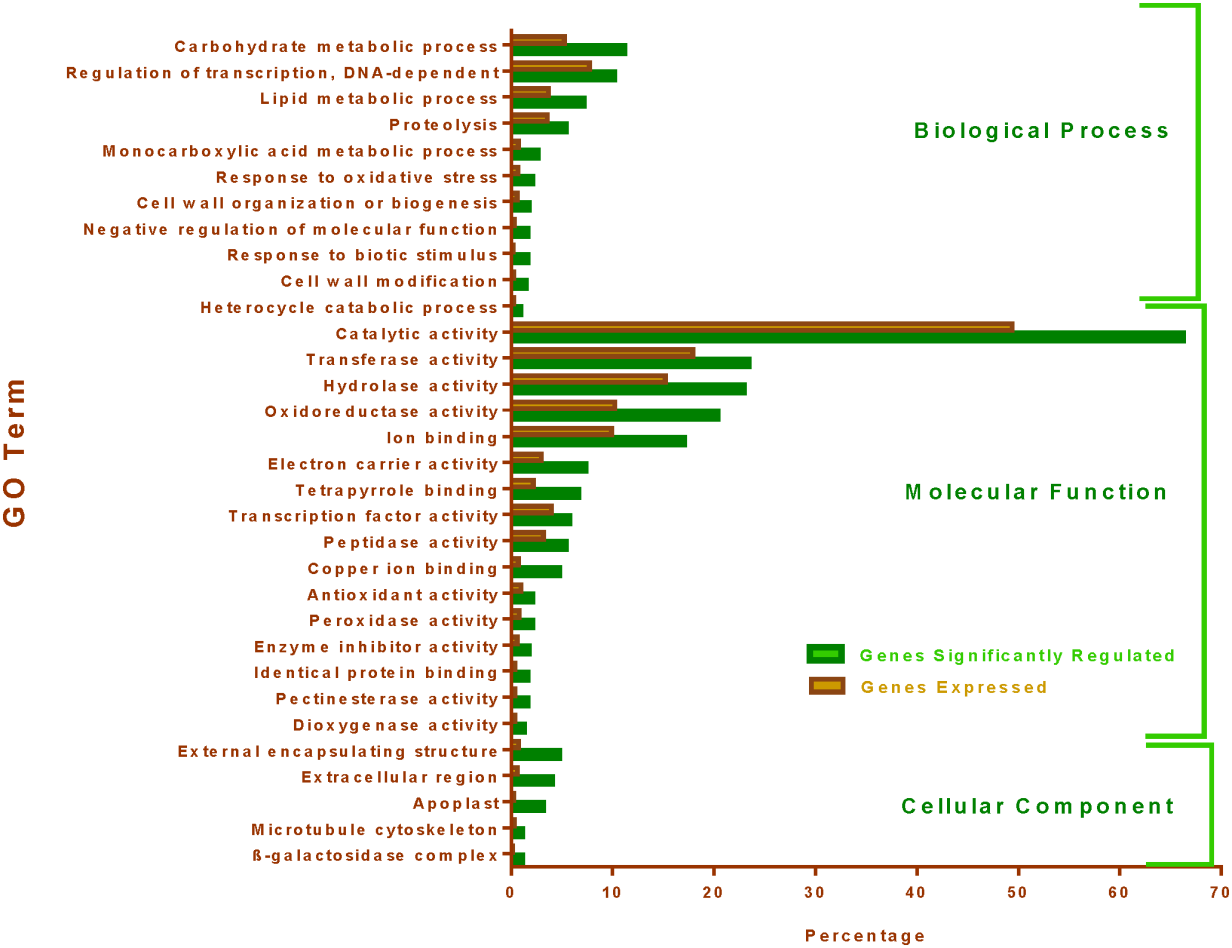
GO terms significantly over-represented (higher percentage) in the differentially expressed genes list compared to background/reference genes (all expressed genes) list in PI 416937 under high VPD conditions. Green bars represent percentage of genes belongs to GO categories from total number of up- and down-regulated genes due to high VPD, and red bars indicate percentage of genes belong to GO category from total number of background/reference list in PI 416937.

In contrast to PI 471938 and Hutcheson, genotype PI 416937 differentially regulated 944 genes when VPD was elevated. Of the 944 differentially regulated genes, 425 were up-regulated and 519 genes were down-regulated (Fig 2 and S3 Table). Only one gene, a putative heat shock protein (Glyma09g24410), was down-regulated in response to elevated VPD in both of the slow-wilting genotypes. The ANK (Gm08:6264434—‐6265362) differentially expressed in Hutcheson was also down-regulated in PI416937 in response to increased VPD. Analyses of gene ontology (GO) [12] revealed that many biological processes and cellular and molecular components were enriched in response to changes in VPD in PI 416937 (Fig 4, S4 Table and S3 Fig). Overall, 577 transcripts in the significantly regulated list of 944 transcripts, and 21,058 transcripts from all 39,068 expressed transcripts were annotated using GO analysis. The GO terms represented among all expressed transcripts was compared to GO terms represented among all differentially expressed transcripts using the SEA method. Fig 4 identifies GO terms significantly enriched among differentially regulated genes compared to the all expressed genes. Major biological processes such as carbohydrate metabolism, transcription regulation, lipid metabolism, oxidative stress response, cell wall organization, and cell wall modification genes were enriched in PI 416937 under high VPD conditions. For molecular functions, catalytic activity genes, transferases, hydrolases, heme-binding motifs, transcription factors, peptidases, peroxidases and antioxidants were over-represented. Under high VPD, cellular components, apoplast, microtubule skeleton and β-galactosidase complexes were over-represented (Fig 4). Under all categories, genes related to cell wall organization were preferentially regulated, which indicated that cell wall-related processes may be modified under high VPD conditions in PI 416937.

Recently the differentially regulated leaf genes in PI 416937 in response to conditions meant to simulate drought were reported. In these experiments, plants were removed from soil and left to experience water deprivations for up to 24 hours [9]. This treatment is distinct from our VPD treatments because water is not limiting in the elevated VPD treatments and does not lead to wilting as was observed in the dry down treatments. Of the 944 transcripts we identified as differentially regulated by high VPD in PI 416937, 812 were identified as being expressed in the transcriptome of the leaves from the dry down experiment as expected given we used the same tissues and genotype. However 472 transcripts out of the 812 transcripts were not identified as clearly differentially expressed in the dry-down study. Two hundred ninety-four genes out of 812 were identified as primarily environmentally regulated (no strong genetic component). Only 46 genes showed a clear genotype dependent response (GXE, or G and E) to both high VPD and dry-down, and only half of these responded in the same direction (increase or decrease) between treatments.

Differentially expressed genes in PI 416937 were analyzed by MapMan [13] to gain insight into the physiological effects of high VPD. MapMan allowed exploration of gene categories that are regulated under high VPD with an emphasis on those related to regulation, metabolism, and development and transport responses. Of all 944 differentially expressed genes, 897 genes were mapped on to 28 of 36 BINs (S3 Fig, Table 3). Five hundred seventy genes were categorized into ten informative BINs, while the remaining genes were categorized into uninformative BINs (S3 Fig). Categories affected by high VPD in PI 416937 include cell wall (BIN 10), lipid metabolism (BIN 11), secondary metabolism (BIN 16), hormone (BIN 17), stress (BIN 20), transcription (BIN 27), protein (BIN 29), signaling (BIN 30), development (BIN 33) and transport (BIN 34) (Table 3). Distribution of these differentially regulated genes into functional categories gives insight into the effects of high VPD on the metabolism of PI 416937 and how it deals with VPD and possibly drought.

**Table 3:** List of genes in each BIN used in MapMan.

Bin	Name	Up- regulated	Down- regulated
1	PS	-	3
2	Major CHO metabolism	1	2
3	Minor CHO metabolism	3	3
4	Glycolysis	-	-
5	Fermentation	4	-
6	Gluconeogenese/ glyoxylate cycle	1	-
7	OPP	-	1
8	TCA/org. transformation	-	-
9	Mitochondrial electron transport / ATP synthesis	2	1
10	Cell wall	14	74
11	Lipid metabolism	9	17
12	N-metabolism	1	1
13	Amino acid metabolism	8	1
14	S-assimilation	-	-
15	Metal handling	5	
16	Secondary metabolism	25	12
17	Hormone metabolism	24	16
18	Co-factor and vitamin metabolism	1	2
19	Tetrapyrrole synthesis	-	-
20	Stress	21	16
21	Redox.regulation	2	3
22	Polyamine metabolism	-	1
23	Nucleotide metabolism	-	2
24	Biodegradation of xenobiotics	-	-
25	C1-metabolism	-	-
26	Miscellaneous	72	86
27	RNA	40	39
28	DNA	4	4
29	Protein	28	31
30	Signaling	25	35
31	Cell	1	16
32	Micro RNA, natural antisense	-	-
33	Development	17	14
34	Transport	22	18
35	Not assigned	76	93
36	C4-photosynthesis	-	-

BIN number and name for MapMan metabolic overview visualization showing the differential expression pattern based on the log2 fold change in leaves of PI 416937 from high VPD vs low VPD. Each BIN contains the number of genes over-expressed and repressed.

### Transcription factors and signaling proteins

**Fig 5:**
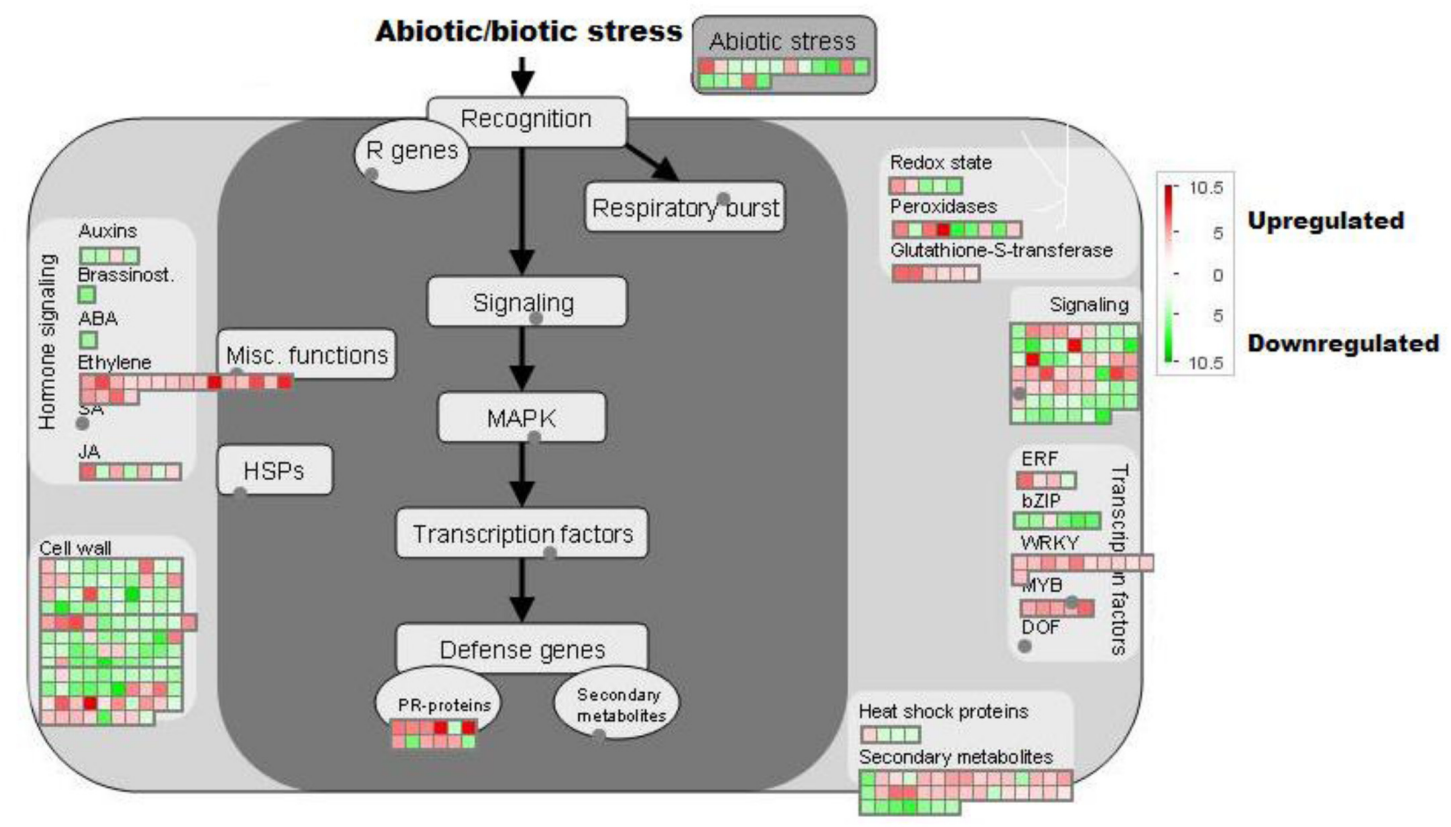
Overview of the stress related genes differentially regulated in PI 416937 exposed to high VPD. Gene transcripts that are induced or repressed due to high VPD are shown in red and green colors, respectively, as shown in the color bar ranging from -10.5 to +10.5.

Several Transcription Factor (TF) genes were differentially regulated in PI 416937 under high VPD conditions relative to low VPD. These TFs play essential roles in regulating stress responsive genes in various signal transduction pathways [14]. Seventy-nine genes annotated as transcription factors were differentially regulated in PI 416937 in response to high VPD; 40 were up-regulated, and 39 were down-regulated. TF genes coding for the zinc-finger family proteins, MYB domain containing family, WRKY, AP2-EREBP/ethylene response factor (ERF), bZIP (Fig 5) and NAC were identified as differentially regulated. The expression of selected transcription factors were validated, including a WRKY, bZIP, NAC and MYB TF (Fig 3A).

Eleven genes belonging to the WRKY transcription factor family were identified as up-regulated under high VPD (Fig 5). Additionally, the level of four transcripts annotated as encoding VQ-motif proteins that are likely to interact with WRKY transcription factors increased in response to high VPD. WRKY transcription factors are known to be involved in both biotic and abiotic stress responses [15,16,17]. The change in expression of several WRKY transcription factor genes in PI 416937 due to high VPD was similar to WRKYs expression pattern in response to other abiotic stresses in numerous plant species. In a study of the soybean genome, 64 *GmWRKY* genes were identified. These WRKY transcription factors conferred salt, cold and drought stress tolerance when expressed in Arabidopsis. [18].

Another important transcription family regulated in this study was bHLH, (basic helix loop helix). The bHLH proteins is a large family of conserved transcription factors that regulate many cellular processes in eukaryotic organisms and are also involved in several responses that are important for plants to cope with drought stress. There are 12 bHLH transcription factors differentially regulated, of which ten are repressed. Among 45 bHLH putative genes in soybean, 14 were determined to be involved in abiotic stress responses [19]. Regulation of bHLH transcription factors due to high VPD in this study also supports a role of these transcription factors in responses to abiotic stresses.

AP2 (APETALA2)–EREBP (ethylene-responsive element binding proteins) transcription factor family also plays a role in plant growth and development, especially in hormonal regulation and abiotic stress responses [18,20]. The present study also showed that transcripts encoding AP2-EREBP (4) family are regulated in response to high VPD.

The levels of seven transcripts annotated as NAC or NAC-like transcription factors increase in expression in response to high VPD in PI 416937. NAC TFs are plant-specific proteins that are known to play a role in response to abiotic stress, including drought [21]. The soybean genome has 152 NAC TFs, 58 of which have been reported as stress responsive [22].

In addition, TFs related to bZIP, Zinc-finger family, MYB and putative DNA-binding are also differentially regulated in this study. Similar transcription factor families were also found to be differentially regulated through microarray assays in soybean genotypes grown under different drought conditions [10]. It was also determined that over-expression or silencing of these gene families played roles in increasing stress tolerance by integrating the responses of other genes[23].

Fifty-nine genes encoding various proteins such as protein kinases, ubiquitins and receptor-like cytoplasmic kinases were abundant among both up-regulated (28) and down-regulated transcripts (31). Genes such as AAA type ATPases and subtilase are mostly involved in either post-translational changes or protein degradation. The mostly up-regulated AAA types of ATPase proteins have chaperon-like activities and play a role in stomatal regulation under dehydration stress [24]. Subtilase serine proteases are involved in the mediation of a signal that controls the formation of guard cells and are involved in the increase of stomatal density [25]. Down-regulation of subtilase-like serine protease in PI 416937 under high VPD might be a signal to form fewer guard cells and decrease stomatal density to control transpiration rate. Indeed, stomatal density is affected by drought stress in the perennial grass *Leymus chinensis* [26]. Measuring guard cell density and size after prolonged exposure of PI 416937 to high VPD could determine if this slow-wilting genotype’s stomatal density is particularly sensitive to high VPD.

Signaling genes (55) include receptor kinases and leucine-rich repeats involved in improving plant performance under drought and also defense mechanisms [27]. It is noteworthy that multiple receptor protein kinase transcripts and leucine-rich repeat transcripts were differentially regulated under high VPD. Leucine-rich repeats are thought to be involved in a large range of abiotic signaling processes by mediating the expression of genes biosynthesizing abscisic acid, peroxidases, catalases and Late Embryogenesis Abundant (LEA) responsive genes[28]. It is not surprising that regulations of suites of genes controlling transcription and signaling are different between the fast and slow-wilting soybean lines. More interesting is that the differentially expressed suite of regulatory genes differs between the two slow-wilting genotypes.

### Hormones

Genes regulating plant hormone signaling pathways were differentially expressed in response to high VPD in PI 416937 (24) (Fig 5). Transcript level changes in response to high VPD indicate that some genes in the abscisic acid, gibberellin, auxin and brassinosteroid related pathways are repressed (16). A transcript encoding a protein responsible for the hydroxylation of abscisic acid was used to validate the transcriptome profiling (Glyma16g08340, Fig 3A). The level of this transcript was substantially increased in PI 416937 under high VPD (Fig 5). Genes involved in the jasmonate pathway have a mixed profile of regulation and may be redirecting jasmonate pathways in response to high VPD. Induction of jasmonic acid biosynthesis genes is an early signal of drought stress in chickpea that conferred better drought tolerance [29].The plant hormone ethylene regulates plant development and responses to various environmental stresses [30]. Fig 5 shows that the level of a suite of genes involved in the ethylene pathway increases in PI 416937 under high VPD. The level of transcripts encoding an ethylene forming enzyme (Glyma08g05500) and an ethylene responsive element (Glyma10g33060) in PI 416937 increased in response to an elevation in VPD (Fig 3A). It is well known that ethylene and abscisic acid are involved in stomatal regulation [31,32]. The response of transcripts associated with phytohormone metabolism in the current study also supports the role of these hormones in the stomatal regulation of PI 416937 exposed to high VPD to control transpiration.

### Cell wall

**Fig 6:**
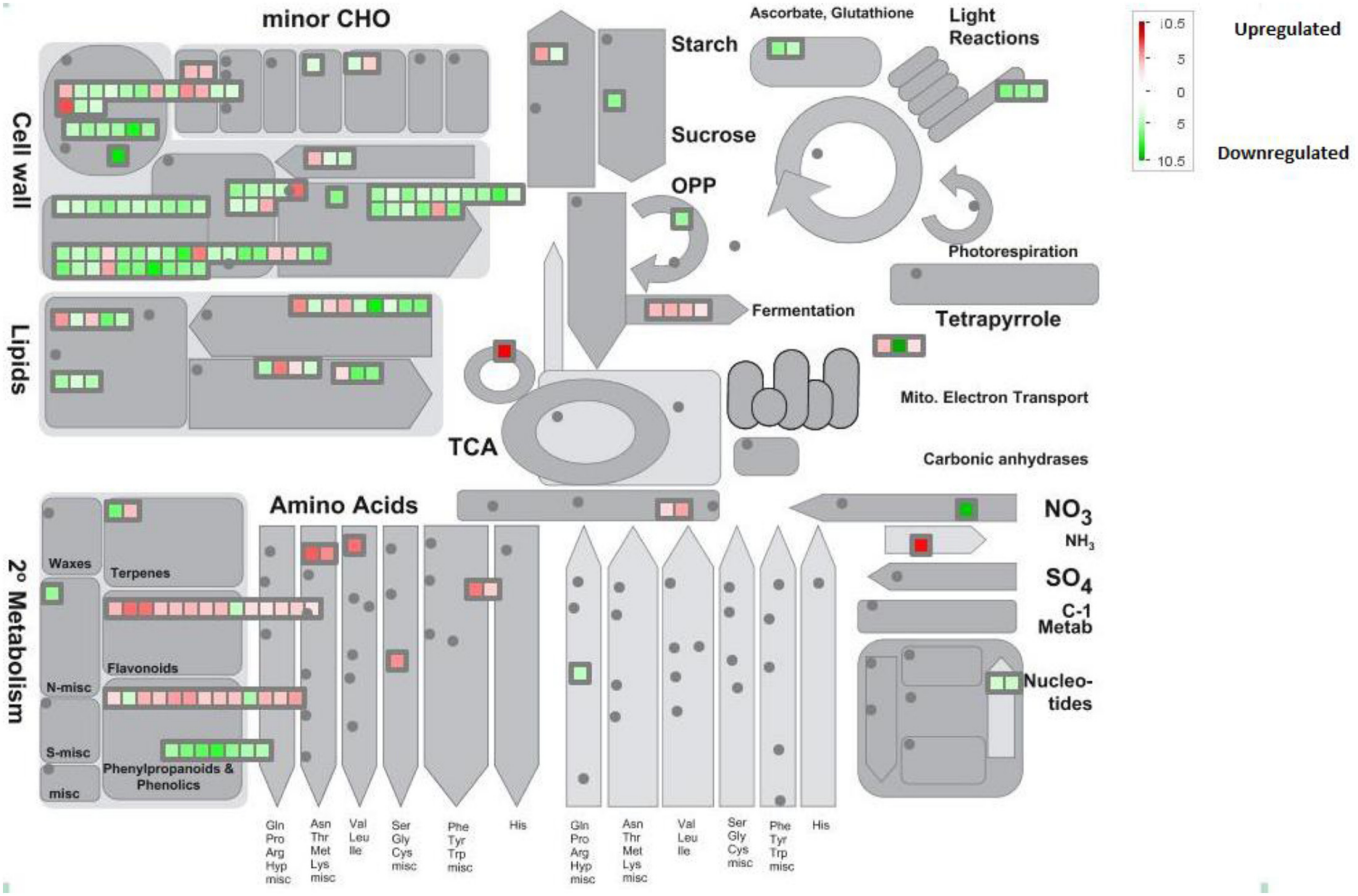
Metabolic overview of genes differentially regulated in PI 416937 due to high VPD. Color bar ranging from green to red represents log2 fold from down-regulated (-10.5) to up-regulated (+10.5) using MapMan.

Eighty-eight genes were assigned to the cell wall BIN, of which, 74 were down-regulated and 14 were up-regulated. Most of these genes are related to cellulose synthesis, cell wall precursors, cell wall modification, pectin esterases, pectate lyases and polygalacturonases (Figs 5 and 6). It is evident from both GO analysis and MapMan that expression of many genes related to the cell wall was sensitive to alterations in VPD. Reduction in the expression of expansin and extensin genes is consistent with the observed reduction in leaf expansion under prolonged high VPD conditions observed in PI 416937 [33]. Decrease in leaf area is a common response to drought in many crops. For example, leaf area decrease was found in drought-tolerant rice cultivar Moroberekan, which showed down-regulation of cell wall depository genes under drought conditions compared to a sensitive variety [34].

### Development

There were 31 genes associated with development that were differentially regulated in response to elevated VPD in PI 416937, 17 were up- and 14 were down- regulated. The genes in the development process are mostly storage proteins such as the LEA. LEA proteins have been reported to stabilize membrane structures [35,36]. The observation that the genes in this category are regulated in both directions may indicate that genes in this category are redirected to deal with high VPD by stabilizing membranes.

### Metabolism

Lipids are the most abundant components of cell membranes and may play roles in resistance of plant cell to environmental stresses [37]. In the present study, regulation of 26 genes involved in lipid metabolism was affected by high VPD in PI 416937. In this category, nine genes decreased in expression, and 17 genes increased in expression in response to elevated VPD. The BIN of lipid metabolism includes genes related to lipid degradation, sphingolipids, fatty acid (FA) synthesis, FA elongation and phospholipid synthesis (Fig 6). In a study with Arabidopsis, polyunsaturated FAs played an important role in responses of photosynthesis and stomatal conductance to environmental stresses such as high VPD [38]. Similarly, changes in gene expression related to lipid metabolism were observed in Arabidopsis due to drought [37]. The mixture of up-regulated and down-regulated genes in response to high VPD may redirect lipid metabolism to help the PI416937 resist high VPD.

Interestingly, there were responses among genes regulating secondary metabolism (37) such as terpene, flavonoid, phenylpropanoid and phenolic biosynthesis (Fig 6). Genes associated with terpene biosynthesis are both up- and down-regulated, possibly indicating a redirection in the terpenes synthesized. An increase in temperature and water deficit caused an emission of terpenes in *Artemisia* spp. plants [39], and changes in gene expression corresponded with simultaneous increases in some terpenes in drought-resistant shrubs [40]. There is a general increase in the genes involved in flavonoid synthesis. Flavonoids accumulate rapidly and play a protective role when plants are exposed to drought stress conditions by scavenging the ROS induced by the stress and also may act as signaling molecules in signaling cascades [41]. Up-regulation of most of the flavonoid genes in the present study was consistent with results of other studies in wheat, rice and potato after exposure to drought stress [42,43,44]. Transcripts involved in phenylpropanoids and phenolics pathways are primarily down-regulated in response to high VPD in PI 416937 (Fig 6). Phenylpropanoids and phenols, derivatives of the shikimate pathway, are modified by oxygenases, ligases, oxidoreductases and transferases to generate a large number of secondary metabolites including lignins, suberins and tannins. An increase in these secondary metabolites may contribute to the tolerance of plants towards stress in some species [45]. In rice, phenylpropanoid biosynthesis was enriched under drought, but genes in the pathway were both up- and down-regulated indicating differential regulation of the enzymes resulting in synthesis of different end-products [46]. Phenols, which are involved in light absorption and neutralization of ROS [47,48], were down-regulated in the present study in contrast with numerous other studies where an increase in total phenolic content in response to drought stress was observed [49,50]. However, the total phenolic compounds estimated in a drought-tolerant winter triticale cultivar showed either no increase or even a small decrease at the expense of synthesizing other components consistent with this study [51]. The unexpected responses of the phenylpropanoid and phenol biosynthesis pathways may reflect the fact that a VPD was imposed in the absence of water limitation. If stomata are more likely to be closed to conserve water, these compounds may be in less demand. Indeed the down-regulation of the light reaction in PI 416937 under high VPD observed in Fig 6 is consistent with limited carbon dioxide availability due to stomatal closure.

Other pathways also appear to be regulated by high VPD in PI 416937; though, due to lower numbers of differentially regulated genes, their interpretation is less certain (Fig 6). Nitrogen metabolic pathways of aromatic amino acid biosynthesis and metabolism of aspartate, serine, glycine, cysteine, alanine and tryptophan were found in up-regulated gene sets. The aromatic amino acids are also produced through the shikimate pathway and play crucial roles in plant growth, development, reproduction and environmental stress response [52,53]. Our study indicated down-regulation of starch and sucrose metabolism, and recent reports indicate a reduction in starch biosynthesis and accumulation, as well as an increased consumption of storage substances under drought [43,54]. Starch and sugar accumulation could be measured under high VPD to determine if accumulation of these essential metabolites is particularly sensitive to water stress in PI 416937.

### Transport

Several transport-related transcripts were differentially regulated under high VPD conditions inPI 416937, most of which were induced (22). Up-regulated transporter transcripts target nitrate, amino acids, ABC transporters, anions, cations, oligopeptides and phosphates. In contrast to the transcripts of these transporters, many of the down-regulated transporters are major intrinsic proteins that act as water channels. The regulation of these water channels in response to elevated VPD may help limit the uptake of water, conserving it for later availability. Aquaporins are involved in water transport and are thought to be involved in maintaining plant homeostasis. Several studies have established their role in response to various abiotic stresses: especially plasma membrane intrinsic proteins and tonoplast intrinsic proteins play major roles in water transport and are responsive to different environmental conditions including VPD [55,56]. A study with transgenic tobacco over-expressing aquaporins showed decreased stomatal conductance at high VPD [57]. Plant cells respond to drought by closing aquaporins through dephosphorylation [58]. Aquaporins become dephosphorylated which lowers water permeability when the plant cell senses lower water potential in the apoplast [59]. Based on GO analysis, it is notable that apoplastic genes are significantly over-represented at elevated VPD in PI 416937 (Fig 4). There are 13 apoplast-related genes down-regulated and 5 up-regulated (S3 and S4 Tables). This further indicated that PI 416937 has a distinctive transport regulatory mechanism that undergoes modification to control water transport under dry environmental conditions. It is also evident from the lack of chemical inhibition of aquaporins in PI 416937 using silver ions that this genotype is different from fast-wilting genotypes [60]. Although silver can inhibit the aquaporins, it is also a potent inhibitor of ethylene action and could disrupt K+ homeostasis and cellular integrity [61,62]. Further investigation related to detailed aquaporin composition in PI 416937 both in roots and leaves will be helpful in understanding the mechanism and role of water transport under elevated VPD levels.

## Conclusion

Our results confirmed that high VPD triggers a novel water-conserving transpiration response in PI 416937 and demonstrated that this response was associated with the differential regulation of large numbers of genes. In contrast, the other two soybean genotypes in the study did not exhibit this novel physiological response to VPD, nor did they exhibit the extensive gene regulation found in PI 416937. Transcriptome analysis of genes differentially expressed under high VPD in PI 416937 shows the large quantitative change leads to changes in pathways related to signaling, including TF, signaling proteins and phytohormone metabolism. The changes of signaling pathways in response to high VPD in the slow-wilting accession PI 416937 target changes in processes associated with cell wall development, metabolism and transport, thus highlighting adaptation of PI 416937 to stress. The signals causing changes in cell wall and stomata development possibly affect leaf expansion and stomatal density. Additionally, changes in expression of LEAs and lipid metabolism specific to PI 416937 may stabilize membranes and redirect secondary metabolites to deal with elevated VPD. Particularly interesting are the decreased levels of transcripts encoding water channel proteins. An early response to high VPD by lowering aquaporin levels may reduce demand for water in PI 416937 before the other two genotypes sense a water deficit, resulting in low hydraulic conductivity and water conservation for sustained growth later in the growing season.

## Supporting Information

S1 Fig Image of the equipment used to induce and measure VPD. (DOCX)

S2 Fig QRT-PCR validation using three replicates for transcriptome study. Genes from A to E were selected from significantly expressed category of PI 416937 and F from PI 471938 for the validation. The X-axis represents soybean genotype and the Y-axis is log2 fold change of transcript levels at high VPD in comparison to low VPD. The first bars represents gene expression data from the transcriptome study in PI 416937 and PI 471938, and remaining bars indicate data from QRT-PCR study. Error bars represent standard deviation from three biological replicates.
(DOCX)

S3 Fig Figure showing overview of differentially regulated genes of PI 416937 in response to high VPD environment. Gene transcripts that are induced or repressed due to high VPD are shown in red and green colors, respectively, as shown in the color bar ranging from -10.5 to +10.5. Genes related to various metabolic processes are grouped under 36 BINS and mapped using MapMan to show different functional categories. Names of different BINS are available in Table 3.
(DOCX)

S1 Table Table showing total sequence and alignment rate of soybean genotypes under low and high VPD treatment using HiSeq. (DOCX)

S2 Table Slopes of TR ± S.E. and R^2^ from linear regression for Hutcheson and PI 471938 when exposed to low, medium and high VPDS in Experiment 1 (EXP 1) and Experiment 2 (EXP 2). Slope 1± S.E., X_0_, Slope 2± S.E. and R^2^ from two-segmental regression for PI 416937 from Experiment 1 and 2. The range of temperature (°C) obtained in each experiment for each genotype was also included.
(DOCX)

S3 Table Table showing genes differentially expressed in PI 416937 and their Q values. Differentially expressed genes in PI 416937 when exposed to high VPD compared to low VPD conditions.
(DOCX)

S4 Table Table listing functional GOs over-represented from the list of differentially expressed genes in PI 416937 at elevated VPD levels. Table includes GO categories with FDR <0.05.
(DOCX)
