# Supplementary figures and images for: Comparisons of the Effects of Elevated Vapor Pressure Deficit on Gene Expression in Leaves among Two Fast-Wilting and a Slow-Wilting Soybean
Source: PLoS One. 2015 Oct 1;10(10):e0139134. doi: 10.1371/journal.pone.0139134 (PMC4591296; doi:10.1371/journal.pone.0139134)

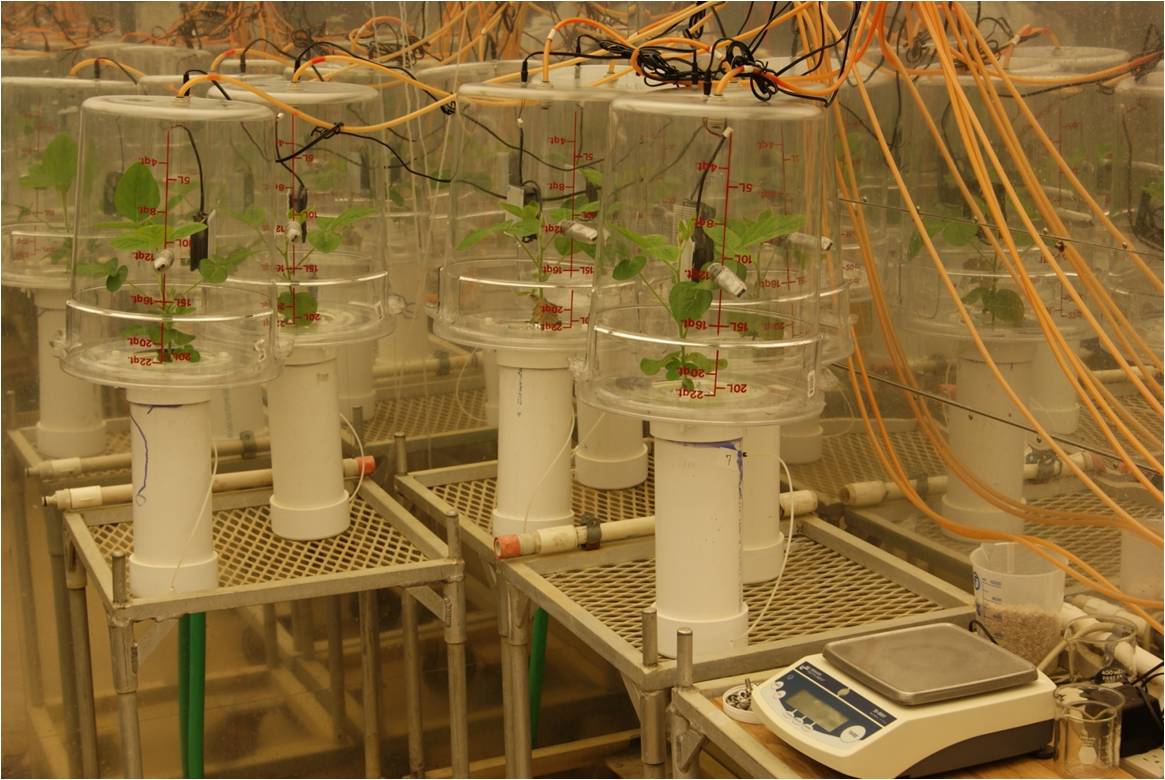


**Supporting Figure 1:**

Image of the equipment used to induce and measure VPD

Supplement: S1 Fig — (DOCX) [file pone.0139134.s001.docx]
