# Supplementary material for: Comparisons of the Effects of Elevated Vapor Pressure Deficit on Gene Expression in Leaves among Two Fast-Wilting and a Slow-Wilting Soybean
Source: PLoS One. 2015 Oct 1;10(10):e0139134. doi: 10.1371/journal.pone.0139134 (PMC4591296; doi:10.1371/journal.pone.0139134)
Supplement: S2 Fig — Genes from A to E were selected from significantly expressed category of PI 416937 and F from PI 471938 for the validation. The X-axis represents soybean genotype and the Y-axis is log2 fold change of transcript levels at high VPD in comparison to low VPD. The first bars represents gene expression data from the transcriptome study in PI 416937 and PI 471938, and remaining bars indicate data from QRT-PCR study. Error bars represent standard deviation from three biological replicates. (DOCX) [file pone.0139134.s002.docx]

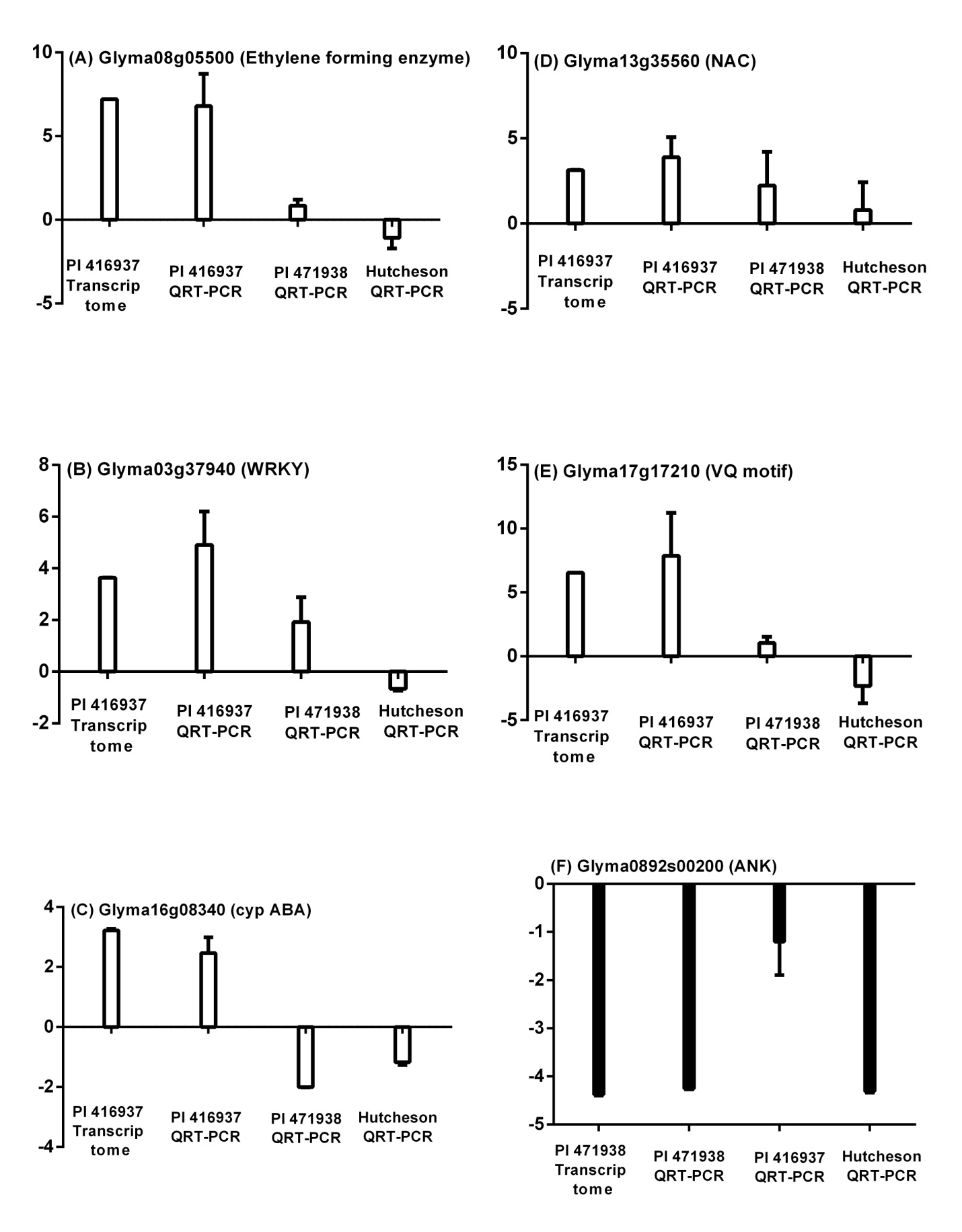


**Supporting Figure 2:**

QRT-PCR validation using three replicates for transcriptome study. Genes from A to E were selected from significantly expressed category of PI 416937 and F from PI 471938 for the validation. The x-axis represents soybean genotype and the y-axis is log2 fold change of transcript levels at high VPD in comparision to low VPD. The first bars represents gene expression data from the transcriptome study in PI 416937 and PI 471938, and remaining bars indicate data from QRT-PCR study. Error bars represent standard deviation from three biological replicates.
