# Supplementary material for: Comparisons of the Effects of Elevated Vapor Pressure Deficit on Gene Expression in Leaves among Two Fast-Wilting and a Slow-Wilting Soybean
Source: PLoS One. 2015 Oct 1;10(10):e0139134. doi: 10.1371/journal.pone.0139134 (PMC4591296; doi:10.1371/journal.pone.0139134)
Supplement: S3 Fig — Gene transcripts that are induced or repressed due to high VPD are shown in red and green colors, respectively, as shown in the color bar ranging from -10.5 to +10.5. Genes related to various metabolic processes are grouped under 36 BINS and mapped using MapMan to show different functional categories. Names of different BINS are available in Table 3. (DOCX) [file pone.0139134.s003.docx]

**
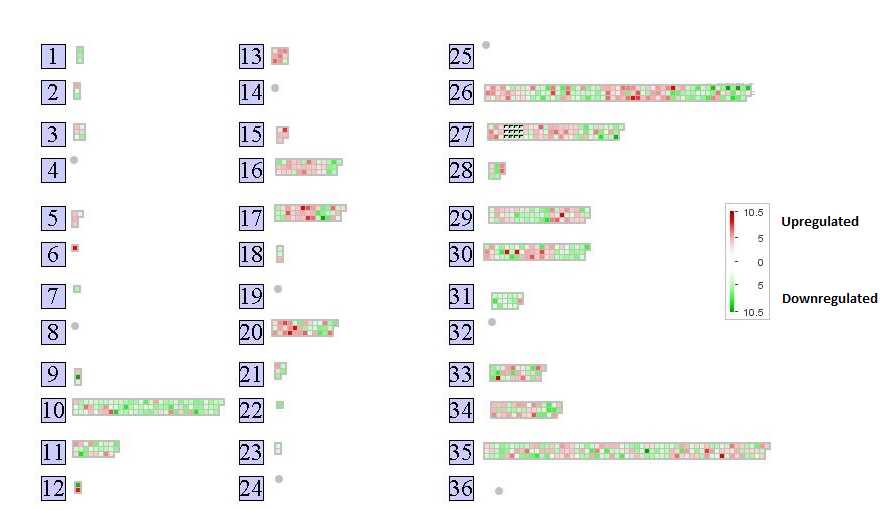
**

**Supporting figure 3**

Figure showing overview of differentially regulated genes of PI 416937 to high VPD environment. Gene transcripts that are induced or repressed due to high VPD are shown in red and green colors, respectively, as shown in the color bar ranging from -10.5 to +10.5. Genes related to various metabolic processes are grouped under 36 BINS and mapped using Mapman to show different functional categories. Names of different BINS are available in table 4.
