# Supplementary material for: Comparisons of the Effects of Elevated Vapor Pressure Deficit on Gene Expression in Leaves among Two Fast-Wilting and a Slow-Wilting Soybean
Source: PLoS One. 2015 Oct 1;10(10):e0139134. doi: 10.1371/journal.pone.0139134 (PMC4591296; doi:10.1371/journal.pone.0139134)
Supplement: S1 Table — (DOCX) [file pone.0139134.s004.docx]

**Supporting table 1.** Table showing total sequence and alignment rate of soybean genotypes under low and high VPD treatment using Hiseq.

| **Sample** | **Treatment** | **Total sequences** | **Alignment rate** |
| --- | --- | --- | --- |
| Hutcheson | high vpd | 12963912 | 82.68% |
| Hutcheson | high vpd | 12471960 | 77.32% |
| Hutcheson | low vpd | 16202795 | 82.32% |
| Hutcheson | low vpd | 17692175 | 83.29% |
| PI471938 | high vpd | 18553692 | 81.10% |
| PI471938 | high vpd | 15949249 | 77.21% |
| PI471938 | low vpd | 18933088 | 80.57% |
| PI471938 | low vpd | 14847930 | 82.89% |
| PI416937 | high vpd | 12187903 | 82.52% |
| PI416937 | high vpd | 13574716 | 76.68% |
| PI416937 | low vpd | 16144905 | 78.87% |
| PI416937 | low vpd | 13572554 | 82.61% |
