# Supplementary material for: Comparisons of the Effects of Elevated Vapor Pressure Deficit on Gene Expression in Leaves among Two Fast-Wilting and a Slow-Wilting Soybean
Source: PLoS One. 2015 Oct 1;10(10):e0139134. doi: 10.1371/journal.pone.0139134 (PMC4591296; doi:10.1371/journal.pone.0139134)
Supplement: S2 Table — Slope 1± S.E., X0, Slope 2± S.E. and R2 from two-segmental regression for PI 416937 from Experiment 1 and 2. The range of temperature (°C) obtained in each experiment for each genotype was also included. (DOCX) [file pone.0139134.s005.docx]

|  | **Slope 1** | | **X_o_** | | **Slope 2** | | **R^2^** | | **Temp (^o^C)** | |
| --- | --- | --- | --- | --- | --- | --- | --- | --- | --- | --- |
|  | **EXP 1** | **EXP 2** | **EXP 1** | **EXP 2** | **EXP 1** | **EXP 2** | **EXP 1** | **EXP 2** | **EXP 1** | **EXP 2** |
| **Hutcheson** | 26.7±0.92 | 27.8±0.97 | _ | _ | _ | _ | 0.95 | 0.97 | 28-34 | 29-34 |
| **PI 471938** | 24.3±1.45 | 22.8±1.22 | _ | _ | _ | _ | 0.94 | 0.96 | 26-34 | 27-34 |
| **PI 416937** | 22.7±0.99 | 21.7±1.12 | 2.20 | 1.99 | 3.41±0.72 | -6.58± 2.17 | 0.88 | 0.86 | 28-34 | 27-34 |

**Supporting Table 2:** Slopes of TR, break point (X_0_), R^2^ and range of temperature (^o^C) obtained for soybean genotypes when exposed to different VPDS in experiment 1 (EXP 1) and experiment 2 (EXP 2)
