# Supplementary material for: Comparisons of the Effects of Elevated Vapor Pressure Deficit on Gene Expression in Leaves among Two Fast-Wilting and a Slow-Wilting Soybean
Source: PLoS One. 2015 Oct 1;10(10):e0139134. doi: 10.1371/journal.pone.0139134 (PMC4591296; doi:10.1371/journal.pone.0139134)
Supplement: S3 Table — Differentially expressed genes in PI 416937 when exposed to high VPD compared to low VPD conditions. (DOCX) [file pone.0139134.s006.docx]

**Supporting Table 3.**

Table showing differentially expressed genes in PI 416937 and their Q values. Differentially expressed genes in PI 416937 when exposed to high VPD compared to low VPD conditions.

| GeneID | log2 | Q |
| --- | --- | --- |
| Glyma10g42340 | 9.5 | 0.009 |
| Glyma18g05890 | 9.1 | 0.000 |
| Glyma18g44310 | 9.0 | 0.015 |
| Glyma02g37990 | 9.0 | 0.000 |
| Glyma02g13810 | 8.7 | 0.022 |
| Glyma17g13730 | 8.7 | 0.001 |
| Glyma11g09880 | 8.6 | 0.008 |
| Glyma12g36220 | 8.6 | 0.000 |
| Glyma02g07940 | 8.3 | 0.000 |
| Glyma10g33060 | 7.9 | 0.001 |
| Glyma03g39050 | 7.7 | 0.001 |
| Glyma03g29950 | 7.7 | 0.003 |
| Glyma20g36160 | 7.6 | 0.011 |
| Glyma11g32070 | 7.4 | 0.047 |
| Glyma02g17490 | 7.4 | 0.027 |
| Gm11:18768533 | 7.3 | 0.000 |
| Glyma15g06780 | 7.3 | 0.000 |
| Glyma11g32500 | 7.2 | 0.002 |
| Glyma08g05500 | 7.2 | 0.000 |
| Glyma10g00980 | 7.2 | 0.007 |
| Glyma15g01390 | 7.2 | 0.000 |
| Glyma11g06690 | 7.2 | 0.000 |
| Glyma11g10080 | 7.1 | 0.024 |
| Glyma05g29440 | 7.1 | 0.050 |
| Gm14:4044040 | 7.0 | 0.000 |
| Glyma19g01470 | 6.9 | 0.000 |
| Glyma15g16790 | 6.9 | 0.000 |
| Glyma10g44160 | 6.9 | 0.013 |
| Glyma14g37440 | 6.8 | 0.000 |
| Glyma01g06150 | 6.7 | 0.018 |
| Glyma12g32230 | 6.7 | 0.043 |
| Glyma08g20230 | 6.6 | 0.000 |
| Glyma15g04620 | 6.6 | 0.016 |
| Glyma10g33650 | 6.6 | 0.009 |
| Glyma20g23420 | 6.5 | 0.000 |
| Glyma19g43820 | 6.5 | 0.030 |
| Glyma17g17210 | 6.5 | 0.000 |
| Glyma07g37240 | 6.5 | 0.000 |
| Glyma08g26840 | 6.5 | 0.016 |
| Glyma17g35110 | 6.5 | 0.000 |
| Glyma03g38570 | 6.5 | 0.001 |
| Glyma17g34530 | 6.5 | 0.024 |
| Glyma18g51680 | 6.5 | 0.001 |
| Glyma15g11220 | 6.4 | 0.031 |
| Glyma06g14680 | 6.4 | 0.004 |
| Glyma08g12400 | 6.4 | 0.023 |
| Glyma19g03730 | 6.4 | 0.000 |
| Glyma02g12220 | 6.4 | 0.004 |
| Glyma19g31580 | 6.4 | 0.000 |
| Glyma05g34660 | 6.3 | 0.025 |
| Glyma09g41440 | 6.3 | 0.035 |
| Glyma07g30500 | 6.3 | 0.007 |
| Glyma02g05880 | 6.3 | 0.014 |
| Glyma03g33110 | 6.3 | 0.006 |
| Glyma11g14260 | 6.3 | 0.002 |
| Glyma18g53080 | 6.3 | 0.015 |
| Glyma18g50320 | 6.3 | 0.000 |
| Glyma09g41620 | 6.3 | 0.002 |
| Gm13:6450883 | 6.3 | 0.002 |
| Glyma11g10600 | 6.3 | 0.000 |
| Glyma16g28500 | 6.2 | 0.025 |
| Glyma15g18210 | 6.2 | 0.025 |
| Glyma11g13850 | 6.2 | 0.033 |
| Glyma07g37280 | 6.1 | 0.000 |
| Glyma17g03350 | 6.1 | 0.019 |
| Glyma18g46500 | 6.1 | 0.020 |
| Glyma18g06360 | 6.1 | 0.024 |
| Glyma17g37790 | 6.1 | 0.002 |
| Glyma01g42430 | 6.1 | 0.050 |
| Glyma11g31150 | 6.0 | 0.001 |
| Glyma07g08090 | 6.0 | 0.001 |
| Glyma12g08520 | 6.0 | 0.004 |
| Glyma04g39860 | 6.0 | 0.050 |
| Glyma13g38240 | 6.0 | 0.000 |
| Glyma02g11630 | 6.0 | 0.000 |
| Glyma01g31750 | 6.0 | 0.000 |
| Gm18:7497194 | 5.9 | 0.010 |
| Glyma01g36590 | 5.9 | 0.000 |
| Glyma10g37940 | 5.9 | 0.046 |
| Glyma0363s00210 | 5.9 | 0.011 |
| Glyma06g15410 | 5.9 | 0.000 |
| Gm07:42390574 | 5.8 | 0.001 |
| Glyma01g33150 | 5.8 | 0.039 |
| Glyma03g22510 | 5.8 | 0.000 |
| Glyma07g32650 | 5.8 | 0.000 |
| Glyma12g15870 | 5.8 | 0.000 |
| Glyma02g35210 | 5.7 | 0.009 |
| Glyma03g03560 | 5.7 | 0.000 |
| Glyma18g06840 | 5.7 | 0.002 |
| Glyma06g17620 | 5.7 | 0.030 |
| Glyma14g00750 | 5.7 | 0.001 |
| Glyma11g31120 | 5.7 | 0.011 |
| Glyma06g12900 | 5.7 | 0.040 |
| Glyma19g26400 | 5.6 | 0.000 |
| Glyma09g23140 | 5.6 | 0.021 |
| Glyma08g44700 | 5.6 | 0.031 |
| Glyma19g31590 | 5.6 | 0.001 |
| Glyma10g30130 | 5.6 | 0.042 |
| Glyma18g48910 | 5.6 | 0.009 |
| Gm04:46760631 | 5.6 | 0.000 |
| Glyma17g03330 | 5.6 | 0.000 |
| Glyma12g07450 | 5.5 | 0.041 |
| Glyma13g40830 | 5.5 | 0.003 |
| Glyma17g18230 | 5.5 | 0.000 |
| Gm18:49342631 | 5.5 | 0.002 |
| Glyma08g44760 | 5.5 | 0.039 |
| Glyma07g23480 | 5.5 | 0.001 |
| Glyma18g16490 | 5.5 | 0.000 |
| Glyma02g06140 | 5.4 | 0.031 |
| Glyma01g34830 | 5.4 | 0.005 |
| Glyma16g07520 | 5.4 | 0.006 |
| Glyma15g11410 | 5.4 | 0.000 |
| Glyma03g37970 | 5.4 | 0.005 |
| Glyma16g28520 | 5.4 | 0.001 |
| Glyma10g04190 | 5.4 | 0.001 |
| Glyma11g05530 | 5.3 | 0.001 |
| Glyma13g17730 | 5.3 | 0.000 |
| Glyma06g10970 | 5.3 | 0.001 |
| Glyma08g27070 | 5.3 | 0.001 |
| Glyma17g07470 | 5.3 | 0.003 |
| Glyma19g32650 | 5.3 | 0.003 |
| Glyma03g37310 | 5.3 | 0.026 |
| Glyma17g03340 | 5.3 | 0.000 |
| Glyma12g02250 | 5.2 | 0.009 |
| Glyma02g43580 | 5.2 | 0.000 |
| Glyma03g30420 | 5.2 | 0.001 |
| Glyma01g38590 | 5.2 | 0.000 |
| Glyma14g08810 | 5.2 | 0.000 |
| Glyma15g06800 | 5.2 | 0.000 |
| Glyma10g03080 | 5.2 | 0.009 |
| Glyma20g27510 | 5.2 | 0.047 |
| Glyma20g29860 | 5.2 | 0.000 |
| Glyma19g43110 | 5.2 | 0.002 |
| Glyma02g06150 | 5.2 | 0.010 |
| Glyma07g05480 | 5.2 | 0.000 |
| Glyma12g36310 | 5.2 | 0.023 |
| Glyma04g08410 | 5.1 | 0.015 |
| Glyma08g43350 | 5.1 | 0.023 |
| Glyma03g28870 | 5.1 | 0.002 |
| Glyma05g21360 | 5.1 | 0.018 |
| Glyma05g02730 | 5.1 | 0.000 |
| Glyma06g45020 | 5.1 | 0.001 |
| Glyma12g04630 | 5.1 | 0.013 |
| Glyma16g26240 | 5.1 | 0.025 |
| Glyma13g03510 | 5.1 | 0.024 |
| Glyma03g22560 | 5.1 | 0.000 |
| Glyma07g38110 | 5.0 | 0.000 |
| Glyma03g24610 | 5.0 | 0.008 |
| Glyma05g35050 | 5.0 | 0.002 |
| Glyma18g44250 | 5.0 | 0.004 |
| Glyma08g46670 | 5.0 | 0.043 |
| Glyma04g36480 | 5.0 | 0.001 |
| Glyma11g19920 | 5.0 | 0.001 |
| Glyma16g29320 | 5.0 | 0.008 |
| Glyma20g34670 | 5.0 | 0.000 |
| Glyma16g08470 | 4.9 | 0.001 |
| Glyma06g38410 | 4.9 | 0.000 |
| Glyma15g40510 | 4.9 | 0.001 |
| Glyma01g40320 | 4.9 | 0.003 |
| Glyma08g04080 | 4.9 | 0.002 |
| Glyma17g34870 | 4.9 | 0.025 |
| Glyma07g03920 | 4.9 | 0.000 |
| Glyma07g08040 | 4.9 | 0.002 |
| Glyma09g28620 | 4.9 | 0.002 |
| Glyma14g04710 | 4.8 | 0.017 |
| Glyma05g27970 | 4.8 | 0.019 |
| Glyma02g41180 | 4.8 | 0.000 |
| Glyma18g44450 | 4.8 | 0.027 |
| Glyma18g43570 | 4.8 | 0.039 |
| Glyma16g26050 | 4.8 | 0.003 |
| Glyma19g24160 | 4.8 | 0.009 |
| Gm03:32874094 | 4.7 | 0.006 |
| Glyma12g12260 | 4.7 | 0.001 |
| Glyma18g05260 | 4.7 | 0.003 |
| Glyma09g05700 | 4.7 | 0.026 |
| Glyma14g01660 | 4.7 | 0.019 |
| Glyma07g29650 | 4.7 | 0.012 |
| Glyma04g23120 | 4.7 | 0.004 |
| Glyma08g04670 | 4.7 | 0.001 |
| Glyma15g05520 | 4.6 | 0.001 |
| Glyma08g21790 | 4.6 | 0.001 |
| Glyma08g12590 | 4.6 | 0.027 |
| Glyma01g28850 | 4.6 | 0.005 |
| Glyma10g04210 | 4.6 | 0.000 |
| Glyma13g02230 | 4.6 | 0.001 |
| Glyma07g00900 | 4.6 | 0.044 |
| Glyma10g26690 | 4.6 | 0.001 |
| Glyma11g02390 | 4.6 | 0.009 |
| Glyma02g10490 | 4.6 | 0.045 |
| Glyma07g09570 | 4.6 | 0.003 |
| Glyma01g44210 | 4.6 | 0.001 |
| Glyma12g06300 | 4.6 | 0.001 |
| Glyma04g04240 | 4.6 | 0.013 |
| Glyma14g38920 | 4.6 | 0.003 |
| Glyma13g44210 | 4.5 | 0.011 |
| Glyma19g39920 | 4.5 | 0.004 |
| Glyma11g13290 | 4.5 | 0.021 |
| Glyma17g35430 | 4.5 | 0.004 |
| Glyma08g10950 | 4.5 | 0.004 |
| Glyma06g16040 | 4.5 | 0.030 |
| Glyma11g08770 | 4.5 | 0.001 |
| Glyma15g04320 | 4.5 | 0.009 |
| Glyma03g00830 | 4.5 | 0.025 |
| Glyma14g00570 | 4.5 | 0.001 |
| Glyma10g37410 | 4.5 | 0.001 |
| Glyma01g00730 | 4.5 | 0.014 |
| Glyma04g37260 | 4.5 | 0.012 |
| Glyma02g11610 | 4.5 | 0.005 |
| Glyma06g47460 | 4.5 | 0.032 |
| Glyma04g02560 | 4.5 | 0.008 |
| Glyma06g11190 | 4.5 | 0.003 |
| Glyma09g04520 | 4.5 | 0.001 |
| Glyma01g43880 | 4.5 | 0.001 |
| Glyma01g37950 | 4.5 | 0.009 |
| Glyma13g43800 | 4.4 | 0.003 |
| Glyma19g34710 | 4.4 | 0.004 |
| Glyma12g00960 | 4.4 | 0.017 |
| Glyma12g07370 | 4.4 | 0.038 |
| Glyma05g32850 | 4.4 | 0.000 |
| Glyma11g20720 | 4.4 | 0.021 |
| Glyma11g16120 | 4.4 | 0.005 |
| Glyma11g37930 | 4.4 | 0.004 |
| Glyma08g40440 | 4.4 | 0.000 |
| Glyma13g06230 | 4.4 | 0.000 |
| Glyma14g05610 | 4.4 | 0.048 |
| Glyma13g35260 | 4.4 | 0.013 |
| Glyma08g24420 | 4.4 | 0.002 |
| Glyma07g34870 | 4.3 | 0.013 |
| Glyma17g03030 | 4.3 | 0.035 |
| Glyma09g28750 | 4.3 | 0.000 |
| Glyma19g41630 | 4.3 | 0.002 |
| Glyma20g16600 | 4.3 | 0.044 |
| Glyma05g28610 | 4.3 | 0.032 |
| Glyma06g18890 | 4.3 | 0.041 |
| Glyma14g05350 | 4.3 | 0.025 |
| Glyma10g13700 | 4.3 | 0.003 |
| Glyma04g41990 | 4.3 | 0.003 |
| Glyma20g00940 | 4.3 | 0.025 |
| Glyma19g37340 | 4.3 | 0.006 |
| Glyma12g02410 | 4.3 | 0.004 |
| Glyma01g04380 | 4.2 | 0.011 |
| Glyma03g02410 | 4.2 | 0.012 |
| Glyma18g44560 | 4.2 | 0.000 |
| Glyma06g40560 | 4.2 | 0.013 |
| Glyma15g40260 | 4.2 | 0.030 |
| Glyma06g14880 | 4.2 | 0.002 |
| Glyma13g26110 | 4.2 | 0.025 |
| Glyma08g18470 | 4.2 | 0.005 |
| Glyma06g01550 | 4.2 | 0.046 |
| Glyma08g01430 | 4.2 | 0.004 |
| Glyma13g18410 | 4.2 | 0.007 |
| Glyma06g47560 | 4.2 | 0.002 |
| Glyma12g35290 | 4.2 | 0.001 |
| Glyma16g06740 | 4.2 | 0.039 |
| Glyma13g29760 | 4.2 | 0.004 |
| Glyma16g05880 | 4.2 | 0.046 |
| Glyma20g34570 | 4.2 | 0.005 |
| Glyma13g32160 | 4.2 | 0.002 |
| Glyma18g48420 | 4.2 | 0.004 |
| Glyma10g37560 | 4.2 | 0.004 |
| Glyma08g19170 | 4.1 | 0.001 |
| Glyma01g32450 | 4.1 | 0.025 |
| Glyma18g47250 | 4.1 | 0.013 |
| Glyma08g11610 | 4.1 | 0.047 |
| Gm20:36804749 | 4.1 | 0.004 |
| Glyma19g03010 | 4.1 | 0.004 |
| Glyma13g20320 | 4.1 | 0.004 |
| Glyma11g09690 | 4.1 | 0.004 |
| Glyma16g23140 | 4.1 | 0.012 |
| Glyma04g42960 | 4.1 | 0.005 |
| Glyma17g07190 | 4.1 | 0.007 |
| Glyma12g01780 | 4.1 | 0.009 |
| Glyma13g36110 | 4.0 | 0.009 |
| Glyma13g38630 | 4.0 | 0.005 |
| Glyma09g05440 | 4.0 | 0.011 |
| Glyma03g04920 | 4.0 | 0.001 |
| Glyma05g25690 | 4.0 | 0.004 |
| Glyma13g23760 | 4.0 | 0.031 |
| Glyma20g38580 | 4.0 | 0.004 |
| Glyma19g36620 | 4.0 | 0.002 |
| Glyma02g40200 | 4.0 | 0.027 |
| Glyma18g49540 | 4.0 | 0.031 |
| Gm08:44738821 | 4.0 | 0.019 |
| Glyma16g01490 | 3.9 | 0.040 |
| Glyma08g07460 | 3.9 | 0.001 |
| Glyma11g37780 | 3.9 | 0.009 |
| Glyma14g26700 | 3.9 | 0.003 |
| Glyma19g40550 | 3.9 | 0.001 |
| Glyma18g17190 | 3.9 | 0.003 |
| Glyma16g01050 | 3.9 | 0.009 |
| Glyma15g42680 | 3.9 | 0.008 |
| Glyma08g11520 | 3.9 | 0.013 |
| Glyma13g24210 | 3.9 | 0.043 |
| Glyma19g40590 | 3.9 | 0.044 |
| Glyma05g37420 | 3.9 | 0.018 |
| Glyma08g47000 | 3.8 | 0.024 |
| Glyma03g30180 | 3.8 | 0.038 |
| Glyma14g09990 | 3.8 | 0.008 |
| Glyma08g39140 | 3.8 | 0.020 |
| Glyma05g08650 | 3.8 | 0.003 |
| Glyma02g14260 | 3.8 | 0.006 |
| Gm08:4443493 | 3.8 | 0.004 |
| Glyma11g18680 | 3.8 | 0.044 |
| Glyma08g18980 | 3.8 | 0.003 |
| Glyma16g28970 | 3.8 | 0.025 |
| Glyma14g01900 | 3.8 | 0.012 |
| Glyma12g22880 | 3.8 | 0.009 |
| Glyma11g18320 | 3.8 | 0.002 |
| Glyma07g05110 | 3.8 | 0.049 |
| Glyma13g07910 | 3.8 | 0.032 |
| Glyma20g26420 | 3.8 | 0.007 |
| Glyma06g39810 | 3.8 | 0.049 |
| Glyma11g29720 | 3.7 | 0.002 |
| Glyma11g11640 | 3.7 | 0.024 |
| Glyma02g09850 | 3.7 | 0.017 |
| Glyma04g01490 | 3.7 | 0.043 |
| Glyma11g00580 | 3.7 | 0.009 |
| Gm20:45262197 | 3.7 | 0.039 |
| Glyma13g28090 | 3.7 | 0.021 |
| Glyma07g31380 | 3.7 | 0.010 |
| Glyma13g09240 | 3.7 | 0.004 |
| Glyma15g18360 | 3.7 | 0.005 |
| Glyma01g01770 | 3.7 | 0.025 |
| Glyma01g43420 | 3.7 | 0.015 |
| Glyma14g05360 | 3.7 | 0.003 |
| Glyma12g12850 | 3.7 | 0.016 |
| Glyma03g41740 | 3.7 | 0.018 |
| Glyma19g41660 | 3.6 | 0.011 |
| Glyma03g37940 | 3.6 | 0.010 |
| Glyma05g38290 | 3.6 | 0.033 |
| Glyma14g07540 | 3.6 | 0.010 |
| Gm19:46515493 | 3.6 | 0.005 |
| Glyma14g00870 | 3.6 | 0.003 |
| Gm05:31906229 | 3.6 | 0.007 |
| Glyma12g35240 | 3.6 | 0.035 |
| Glyma08g07470 | 3.6 | 0.018 |
| Glyma01g37090 | 3.6 | 0.004 |
| Glyma10g29750 | 3.6 | 0.013 |
| Glyma19g03390 | 3.6 | 0.040 |
| Glyma01g44430 | 3.6 | 0.010 |
| Glyma07g05800 | 3.6 | 0.042 |
| Glyma08g16190 | 3.6 | 0.014 |
| Glyma17g03500 | 3.6 | 0.009 |
| Glyma19g37130 | 3.6 | 0.004 |
| Glyma18g08720 | 3.5 | 0.020 |
| Glyma19g40720 | 3.5 | 0.021 |
| Glyma18g41340 | 3.5 | 0.019 |
| Glyma08g26830 | 3.5 | 0.006 |
| Glyma07g04460 | 3.5 | 0.020 |
| Glyma04g09710 | 3.5 | 0.033 |
| Glyma11g35170 | 3.5 | 0.004 |
| Glyma10g39980 | 3.5 | 0.027 |
| Glyma13g26630 | 3.5 | 0.012 |
| Glyma11g08570 | 3.5 | 0.005 |
| Glyma08g14960 | 3.4 | 0.008 |
| Glyma09g09910 | 3.4 | 0.038 |
| Glyma10g08540 | 3.4 | 0.042 |
| Glyma19g09320 | 3.4 | 0.030 |
| Glyma20g36000 | 3.4 | 0.033 |
| Glyma05g04960 | 3.4 | 0.007 |
| Glyma17g34160 | 3.4 | 0.021 |
| Glyma11g02980 | 3.4 | 0.016 |
| Glyma13g03990 | 3.4 | 0.019 |
| Glyma15g01470 | 3.4 | 0.022 |
| Glyma19g31340 | 3.4 | 0.024 |
| Glyma18g41290 | 3.4 | 0.012 |
| Glyma01g26220 | 3.4 | 0.010 |
| Glyma11g07750 | 3.3 | 0.030 |
| Glyma08g41090 | 3.3 | 0.032 |
| Glyma01g42670 | 3.3 | 0.043 |
| Glyma06g13100 | 3.3 | 0.041 |
| Glyma18g45260 | 3.3 | 0.016 |
| Glyma14g38610 | 3.3 | 0.013 |
| Glyma18g50060 | 3.3 | 0.009 |
| Glyma07g25390 | 3.3 | 0.012 |
| Glyma19g44810 | 3.3 | 0.018 |
| Gm03:6920202 | 3.3 | 0.042 |
| Glyma08g07590 | 3.3 | 0.030 |
| Glyma09g34140 | 3.2 | 0.034 |
| Glyma06g15220 | 3.2 | 0.007 |
| Glyma12g30410 | 3.2 | 0.016 |
| Gm03:36679740 | 3.2 | 0.036 |
| Glyma16g08340 | 3.2 | 0.048 |
| Glyma03g04990 | 3.2 | 0.022 |
| Glyma11g32600 | 3.2 | 0.015 |
| Glyma16g29650 | 3.2 | 0.014 |
| Glyma09g01650 | 3.2 | 0.035 |
| Glyma13g06170 | 3.2 | 0.030 |
| Glyma01g43010 | 3.2 | 0.046 |
| Glyma13g35550 | 3.1 | 0.045 |
| Glyma01g24880 | 3.1 | 0.025 |
| Glyma13g35560 | 3.1 | 0.034 |
| Glyma17g23740 | 3.1 | 0.020 |
| Glyma10g00220 | 3.1 | 0.027 |
| Glyma05g24740 | 3.1 | 0.047 |
| Glyma12g30270 | 3.1 | 0.028 |
| Glyma13g35220 | 3.0 | 0.044 |
| Glyma03g34480 | 3.0 | 0.021 |
| Glyma19g39030 | 3.0 | 0.032 |
| Glyma13g36500 | 3.0 | 0.046 |
| Glyma09g30300 | 3.0 | 0.024 |
| Glyma10g37600 | 3.0 | 0.022 |
| Glyma09g05470 | 3.0 | 0.042 |
| Glyma13g16410 | 3.0 | 0.017 |
| Glyma13g37970 | 3.0 | 0.020 |
| Glyma02g02560 | 3.0 | 0.025 |
| Glyma07g16760 | 3.0 | 0.040 |
| Glyma01g42420 | 3.0 | 0.032 |
| Glyma12g13020 | 2.9 | 0.036 |
| Glyma08g18990 | 2.9 | 0.044 |
| Glyma15g00570 | 2.9 | 0.023 |
| Glyma06g01240 | 2.9 | 0.024 |
| Glyma05g29400 | 2.8 | 0.047 |
| Glyma19g30600 | 2.8 | 0.029 |
| Glyma03g39450 | 2.8 | 0.039 |
| Glyma06g19710 | 2.8 | 0.046 |
| Glyma19g44790 | 2.8 | 0.030 |
| Glyma02g03870 | 2.8 | 0.042 |
| Glyma19g02180 | 2.8 | 0.047 |
| Glyma16g03620 | 2.7 | 0.046 |
| Glyma08g12070 | 2.7 | 0.049 |
| Glyma19g37120 | 2.7 | 0.035 |
| Glyma13g44700 | 2.7 | 0.047 |
| Glyma06g09520 | 2.7 | 0.043 |
| Glyma19g00980 | 2.7 | 0.046 |
| Glyma08g44620 | -2.7 | 0.046 |
| Glyma08g15480 | -2.7 | 0.049 |
| Glyma02g00380 | -2.8 | 0.031 |
| Glyma17g37640 | -2.9 | 0.034 |
| Glyma19g36820 | -2.9 | 0.050 |
| Glyma16g27100 | -2.9 | 0.047 |
| Glyma20g19200 | -2.9 | 0.025 |
| Glyma02g03960 | -2.9 | 0.047 |
| Glyma11g36600 | -2.9 | 0.028 |
| Glyma10g03770 | -2.9 | 0.050 |
| Glyma10g05720 | -2.9 | 0.040 |
| Gm20:46584521 | -3.0 | 0.037 |
| Glyma07g08400 | -3.0 | 0.023 |
| Glyma13g32040 | -3.0 | 0.040 |
| Glyma12g33530 | -3.0 | 0.027 |
| Glyma04g34880 | -3.0 | 0.044 |
| Glyma06g26810 | -3.0 | 0.046 |
| Glyma04g41460 | -3.0 | 0.048 |
| Glyma07g15190 | -3.1 | 0.042 |
| Glyma06g35110 | -3.1 | 0.046 |
| Glyma04g37330 | -3.1 | 0.033 |
| Glyma13g27540 | -3.1 | 0.043 |
| Glyma03g35900 | -3.1 | 0.037 |
| Glyma06g02650 | -3.1 | 0.020 |
| Glyma01g23870 | -3.1 | 0.027 |
| Glyma15g07270 | -3.2 | 0.050 |
| Glyma16g18090 | -3.2 | 0.028 |
| Glyma17g11670 | -3.2 | 0.042 |
| Glyma14g20450 | -3.2 | 0.018 |
| Glyma09g05300 | -3.2 | 0.048 |
| Glyma14g00310 | -3.2 | 0.040 |
| Glyma17g08570 | -3.2 | 0.019 |
| Glyma09g42020 | -3.2 | 0.018 |
| Glyma15g43040 | -3.2 | 0.047 |
| Glyma02g10770 | -3.2 | 0.020 |
| Glyma05g01880 | -3.2 | 0.049 |
| Glyma03g31530 | -3.2 | 0.043 |
| Glyma12g04880 | -3.2 | 0.037 |
| Gm17:38902572 | -3.2 | 0.014 |
| Glyma02g47880 | -3.2 | 0.010 |
| Glyma13g10460 | -3.3 | 0.047 |
| Glyma01g40270 | -3.3 | 0.049 |
| Glyma01g03760 | -3.3 | 0.018 |
| Glyma02g05240 | -3.3 | 0.031 |
| Glyma19g27530 | -3.3 | 0.046 |
| Glyma14g10890 | -3.3 | 0.021 |
| Glyma05g01240 | -3.3 | 0.043 |
| Glyma10g01910 | -3.3 | 0.034 |
| Glyma13g03810 | -3.3 | 0.012 |
| Glyma18g42580 | -3.3 | 0.032 |
| Glyma08g44210 | -3.3 | 0.016 |
| Glyma02g11100 | -3.3 | 0.027 |
| Glyma10g34630 | -3.3 | 0.042 |
| Glyma08g06020 | -3.3 | 0.016 |
| Glyma01g45440 | -3.3 | 0.015 |
| Glyma08g03910 | -3.3 | 0.033 |
| Glyma02g42240 | -3.4 | 0.028 |
| Glyma17g04740 | -3.4 | 0.026 |
| Glyma19g34860 | -3.4 | 0.005 |
| Glyma20g24940 | -3.4 | 0.024 |
| Glyma01g00540 | -3.4 | 0.025 |
| Glyma12g35610 | -3.4 | 0.040 |
| Glyma01g42710 | -3.4 | 0.026 |
| Gm04:42438867 | -3.4 | 0.023 |
| Glyma04g11640 | -3.4 | 0.011 |
| Glyma19g28950 | -3.4 | 0.044 |
| Glyma20g04490 | -3.5 | 0.023 |
| Glyma13g42600 | -3.5 | 0.036 |
| Glyma08g04560 | -3.5 | 0.025 |
| Glyma08g05130 | -3.5 | 0.012 |
| Glyma15g20550 | -3.5 | 0.006 |
| Glyma07g06440 | -3.5 | 0.007 |
| Glyma16g03050 | -3.5 | 0.011 |
| Glyma02g45810 | -3.5 | 0.016 |
| Glyma08g27440 | -3.5 | 0.015 |
| Glyma04g05350 | -3.5 | 0.031 |
| Glyma05g22060 | -3.6 | 0.041 |
| Glyma01g36300 | -3.6 | 0.031 |
| Glyma05g33700 | -3.6 | 0.006 |
| Glyma04g13490 | -3.6 | 0.003 |
| Glyma08g15380 | -3.6 | 0.027 |
| Glyma18g00500 | -3.6 | 0.003 |
| Glyma07g32620 | -3.6 | 0.010 |
| Glyma12g03510 | -3.6 | 0.026 |
| Glyma13g36930 | -3.6 | 0.013 |
| Glyma03g23390 | -3.6 | 0.046 |
| Glyma20g27280 | -3.6 | 0.006 |
| Glyma13g29690 | -3.6 | 0.011 |
| Glyma16g17500 | -3.6 | 0.030 |
| Glyma16g27050 | -3.7 | 0.024 |
| Glyma11g01130 | -3.7 | 0.018 |
| Glyma12g35590 | -3.7 | 0.034 |
| Glyma11g04820 | -3.7 | 0.044 |
| Glyma08g01610 | -3.7 | 0.005 |
| Glyma10g32940 | -3.7 | 0.031 |
| Glyma03g04950 | -3.7 | 0.024 |
| Glyma14g01610 | -3.7 | 0.016 |
| Glyma07g03840 | -3.7 | 0.046 |
| Glyma04g35270 | -3.7 | 0.025 |
| Glyma11g15780 | -3.7 | 0.020 |
| Glyma19g36500 | -3.7 | 0.026 |
| Glyma17g02440 | -3.7 | 0.008 |
| Glyma03g31240 | -3.7 | 0.041 |
| Glyma15g43180 | -3.7 | 0.005 |
| Glyma02g04510 | -3.7 | 0.004 |
| Glyma05g36930 | -3.7 | 0.027 |
| Glyma17g13180 | -3.7 | 0.020 |
| Glyma09g24410 | -3.7 | 0.006 |
| Glyma08g11300 | -3.7 | 0.005 |
| Glyma16g29960 | -3.7 | 0.010 |
| Glyma01g36860 | -3.7 | 0.019 |
| Glyma20g29840 | -3.7 | 0.009 |
| Glyma09g07240 | -3.8 | 0.025 |
| Glyma14g35270 | -3.8 | 0.016 |
| Glyma19g43280 | -3.8 | 0.037 |
| Glyma03g03100 | -3.8 | 0.005 |
| Glyma11g19490 | -3.8 | 0.003 |
| Glyma12g01410 | -3.8 | 0.025 |
| Glyma15g02750 | -3.8 | 0.030 |
| Glyma08g22190 | -3.8 | 0.035 |
| Glyma14g06370 | -3.8 | 0.049 |
| Glyma09g03090 | -3.8 | 0.043 |
| Glyma02g15670 | -3.8 | 0.005 |
| Glyma06g47200 | -3.8 | 0.005 |
| Glyma18g07330 | -3.8 | 0.031 |
| Glyma03g33800 | -3.8 | 0.009 |
| Glyma07g04960 | -3.8 | 0.008 |
| Glyma05g28390 | -3.8 | 0.026 |
| Glyma04g04390 | -3.8 | 0.021 |
| Glyma18g25160 | -3.8 | 0.016 |
| Glyma15g09850 | -3.9 | 0.006 |
| Glyma07g01250 | -3.9 | 0.007 |
| Glyma06g02240 | -3.9 | 0.031 |
| Glyma07g01680 | -3.9 | 0.021 |
| Glyma06g09390 | -3.9 | 0.027 |
| Glyma15g13640 | -3.9 | 0.047 |
| Glyma09g35210 | -3.9 | 0.046 |
| Glyma14g07700 | -3.9 | 0.023 |
| Glyma20g03910 | -3.9 | 0.041 |
| Glyma05g00470 | -3.9 | 0.009 |
| Glyma11g36730 | -3.9 | 0.002 |
| Glyma01g29410 | -3.9 | 0.040 |
| Glyma19g36470 | -3.9 | 0.011 |
| Glyma14g09170 | -3.9 | 0.031 |
| Glyma19g33300 | -4.0 | 0.011 |
| Glyma09g15620 | -4.0 | 0.014 |
| Glyma15g17550 | -4.0 | 0.036 |
| Glyma08g45580 | -4.0 | 0.029 |
| Glyma20g27990 | -4.0 | 0.018 |
| Glyma16g04770 | -4.0 | 0.043 |
| Glyma08g45890 | -4.0 | 0.036 |
| Glyma20g11610 | -4.0 | 0.005 |
| Gm10:1409650 | -4.0 | 0.048 |
| Glyma14g02350 | -4.0 | 0.001 |
| Glyma13g04590 | -4.0 | 0.004 |
| Glyma16g07450 | -4.0 | 0.013 |
| Glyma01g37540 | -4.0 | 0.001 |
| Glyma02g40980 | -4.1 | 0.005 |
| Glyma07g33950 | -4.1 | 0.001 |
| Glyma06g12470 | -4.1 | 0.002 |
| Glyma06g06110 | -4.1 | 0.028 |
| Glyma11g08320 | -4.1 | 0.047 |
| Glyma11g06290 | -4.1 | 0.007 |
| Glyma16g02150 | -4.1 | 0.001 |
| Glyma13g31530 | -4.1 | 0.013 |
| Glyma06g42110 | -4.1 | 0.009 |
| Glyma05g03580 | -4.1 | 0.010 |
| Glyma11g02610 | -4.2 | 0.032 |
| Glyma13g28890 | -4.2 | 0.006 |
| Glyma03g41990 | -4.2 | 0.014 |
| Glyma06g19720 | -4.2 | 0.001 |
| Glyma20g02210 | -4.2 | 0.030 |
| Glyma03g01630 | -4.2 | 0.009 |
| Glyma16g02020 | -4.2 | 0.030 |
| Glyma18g38430 | -4.2 | 0.008 |
| Glyma06g02760 | -4.2 | 0.002 |
| Glyma13g21970 | -4.2 | 0.016 |
| Glyma18g04710 | -4.2 | 0.034 |
| Glyma20g34340 | -4.2 | 0.038 |
| Glyma17g14730 | -4.2 | 0.004 |
| Glyma15g12170 | -4.2 | 0.003 |
| Glyma02g07850 | -4.2 | 0.031 |
| Glyma09g40800 | -4.2 | 0.017 |
| Glyma19g41950 | -4.2 | 0.025 |
| Gm15:4071884 | -4.2 | 0.041 |
| Glyma14g00830 | -4.2 | 0.001 |
| Glyma20g10310 | -4.2 | 0.047 |
| Glyma03g03270 | -4.2 | 0.002 |
| Glyma08g05660 | -4.2 | 0.049 |
| Glyma15g42010 | -4.2 | 0.031 |
| Glyma17g35290 | -4.3 | 0.003 |
| Glyma13g02960 | -4.3 | 0.025 |
| Glyma02g00930 | -4.3 | 0.023 |
| Glyma17g02020 | -4.3 | 0.004 |
| Glyma10g31550 | -4.3 | 0.009 |
| Glyma06g00720 | -4.3 | 0.018 |
| Glyma06g19440 | -4.3 | 0.043 |
| Glyma08g17230 | -4.3 | 0.003 |
| Gm14:8789043 | -4.3 | 0.000 |
| Glyma18g08530 | -4.3 | 0.009 |
| Glyma13g35050 | -4.3 | 0.009 |
| Glyma04g00450 | -4.3 | 0.007 |
| Glyma06g04220 | -4.4 | 0.011 |
| Glyma09g36460 | -4.4 | 0.013 |
| Glyma02g07960 | -4.4 | 0.001 |
| Glyma11g34630 | -4.4 | 0.011 |
| Glyma17g04940 | -4.4 | 0.001 |
| Glyma01g38980 | -4.4 | 0.001 |
| Glyma02g47790 | -4.4 | 0.001 |
| Glyma05g34290 | -4.4 | 0.013 |
| Glyma11g37610 | -4.4 | 0.009 |
| Glyma07g32230 | -4.4 | 0.011 |
| Glyma08g19000 | -4.4 | 0.004 |
| Glyma10g39740 | -4.4 | 0.018 |
| Glyma06g04740 | -4.4 | 0.005 |
| Glyma19g25150 | -4.4 | 0.034 |
| Glyma07g07560 | -4.5 | 0.018 |
| Glyma09g03080 | -4.5 | 0.022 |
| Glyma05g30460 | -4.5 | 0.012 |
| Glyma17g05230 | -4.5 | 0.010 |
| Glyma04g11870 | -4.5 | 0.001 |
| Glyma12g17510 | -4.5 | 0.016 |
| Glyma09g35030 | -4.5 | 0.025 |
| Glyma10g39730 | -4.5 | 0.008 |
| Glyma02g12140 | -4.5 | 0.041 |
| Glyma06g04580 | -4.5 | 0.001 |
| Glyma01g20460 | -4.5 | 0.000 |
| Glyma18g04780 | -4.5 | 0.002 |
| Glyma13g26030 | -4.5 | 0.050 |
| Glyma08g05900 | -4.5 | 0.044 |
| Glyma10g28220 | -4.6 | 0.023 |
| Glyma08g07380 | -4.6 | 0.002 |
| Glyma06g11820 | -4.6 | 0.010 |
| Glyma12g06730 | -4.6 | 0.002 |
| Glyma01g40550 | -4.6 | 0.002 |
| Glyma09g30700 | -4.6 | 0.022 |
| Glyma20g16490 | -4.6 | 0.028 |
| Glyma05g28870 | -4.6 | 0.003 |
| Glyma05g34020 | -4.6 | 0.009 |
| Glyma13g05810 | -4.6 | 0.016 |
| Glyma09g18550 | -4.6 | 0.024 |
| Glyma07g09730 | -4.6 | 0.001 |
| Glyma12g06480 | -4.6 | 0.044 |
| Glyma20g32660 | -4.6 | 0.019 |
| Glyma11g18070 | -4.6 | 0.008 |
| Glyma18g08190 | -4.6 | 0.019 |
| Glyma08g28770 | -4.6 | 0.016 |
| Glyma09g36080 | -4.6 | 0.013 |
| Glyma09g40420 | -4.6 | 0.002 |
| Glyma06g17730 | -4.6 | 0.012 |
| Glyma12g10710 | -4.6 | 0.027 |
| Glyma01g44480 | -4.6 | 0.003 |
| Glyma13g43410 | -4.7 | 0.040 |
| Glyma06g16420 | -4.7 | 0.002 |
| Gm17:11677246 | -4.7 | 0.001 |
| Glyma19g02650 | -4.7 | 0.012 |
| Glyma06g05860 | -4.7 | 0.046 |
| Glyma06g23590 | -4.7 | 0.024 |
| Glyma04g03920 | -4.7 | 0.002 |
| Glyma17g13690 | -4.7 | 0.001 |
| Glyma07g16080 | -4.8 | 0.032 |
| Glyma03g30860 | -4.8 | 0.001 |
| Glyma02g12570 | -4.8 | 0.003 |
| Glyma12g10420 | -4.8 | 0.008 |
| Glyma05g04270 | -4.8 | 0.000 |
| Glyma08g45140 | -4.8 | 0.022 |
| Glyma19g32760 | -4.8 | 0.048 |
| Glyma10g00260 | -4.8 | 0.001 |
| Glyma17g17970 | -4.8 | 0.000 |
| Glyma03g42440 | -4.8 | 0.020 |
| Glyma14g20440 | -4.8 | 0.019 |
| Glyma15g14000 | -4.8 | 0.002 |
| Glyma13g43920 | -4.9 | 0.001 |
| Glyma17g35090 | -4.9 | 0.035 |
| Glyma09g04450 | -4.9 | 0.016 |
| Glyma13g21440 | -4.9 | 0.004 |
| Glyma01g42950 | -4.9 | 0.013 |
| Glyma03g41280 | -4.9 | 0.047 |
| Glyma19g43340 | -4.9 | 0.006 |
| Glyma11g02130 | -4.9 | 0.022 |
| Glyma15g29880 | -4.9 | 0.014 |
| Glyma18g07240 | -4.9 | 0.039 |
| Glyma05g31290 | -5.0 | 0.002 |
| Glyma10g24630 | -5.0 | 0.000 |
| Glyma19g28200 | -5.0 | 0.046 |
| Glyma11g05900 | -5.0 | 0.001 |
| Glyma15g01420 | -5.0 | 0.001 |
| Glyma06g12680 | -5.0 | 0.007 |
| Glyma15g09560 | -5.0 | 0.000 |
| Glyma07g05860 | -5.0 | 0.025 |
| Glyma18g18980 | -5.0 | 0.006 |
| Glyma04g17600 | -5.0 | 0.000 |
| Glyma06g04020 | -5.0 | 0.011 |
| Glyma14g09620 | -5.0 | 0.000 |
| Glyma15g08770 | -5.0 | 0.006 |
| Glyma02g39660 | -5.0 | 0.016 |
| Glyma10g00250 | -5.1 | 0.002 |
| Glyma04g23760 | -5.1 | 0.047 |
| Glyma17g16890 | -5.1 | 0.015 |
| Glyma15g15950 | -5.1 | 0.017 |
| Glyma11g00240 | -5.1 | 0.004 |
| Glyma10g32250 | -5.1 | 0.000 |
| Glyma07g15120 | -5.1 | 0.011 |
| Glyma19g40020 | -5.1 | 0.000 |
| Glyma08g17280 | -5.1 | 0.000 |
| Glyma13g42370 | -5.1 | 0.004 |
| Glyma17g35530 | -5.1 | 0.000 |
| Glyma08g03620 | -5.2 | 0.038 |
| Glyma09g32080 | -5.2 | 0.000 |
| Glyma12g32590 | -5.2 | 0.001 |
| Glyma11g26240 | -5.2 | 0.001 |
| Glyma02g11720 | -5.2 | 0.000 |
| Glyma18g00630 | -5.2 | 0.000 |
| Glyma08g45150 | -5.2 | 0.001 |
| Glyma02g32130 | -5.2 | 0.000 |
| Glyma08g11140 | -5.2 | 0.002 |
| Glyma11g18340 | -5.2 | 0.004 |
| Glyma09g33880 | -5.2 | 0.000 |
| Glyma03g38640 | -5.2 | 0.001 |
| Glyma07g04310 | -5.3 | 0.000 |
| Glyma06g46360 | -5.3 | 0.001 |
| Glyma17g11940 | -5.3 | 0.003 |
| Glyma12g35700 | -5.3 | 0.040 |
| Glyma10g12400 | -5.3 | 0.030 |
| Glyma12g34100 | -5.3 | 0.005 |
| Glyma10g33790 | -5.3 | 0.042 |
| Glyma01g05370 | -5.3 | 0.037 |
| Glyma09g37090 | -5.3 | 0.001 |
| Glyma19g05120 | -5.3 | 0.001 |
| Glyma13g38040 | -5.3 | 0.003 |
| Glyma19g30180 | -5.3 | 0.016 |
| Glyma04g02140 | -5.3 | 0.000 |
| Glyma13g34770 | -5.3 | 0.026 |
| Glyma11g07240 | -5.3 | 0.015 |
| Glyma12g14130 | -5.3 | 0.004 |
| Glyma16g12090 | -5.4 | 0.000 |
| Glyma12g28890 | -5.4 | 0.037 |
| Glyma01g39370 | -5.4 | 0.000 |
| Glyma16g33800 | -5.4 | 0.000 |
| Glyma13g42680 | -5.4 | 0.004 |
| Glyma13g29630 | -5.4 | 0.024 |
| Glyma17g38120 | -5.4 | 0.000 |
| Glyma04g08100 | -5.4 | 0.023 |
| Glyma14g35220 | -5.5 | 0.001 |
| Glyma15g37480 | -5.5 | 0.000 |
| Glyma13g17740 | -5.5 | 0.000 |
| Glyma02g08910 | -5.5 | 0.000 |
| Glyma17g35710 | -5.5 | 0.000 |
| Glyma11g10130 | -5.5 | 0.004 |
| Glyma17g31330 | -5.5 | 0.000 |
| Glyma15g41940 | -5.5 | 0.000 |
| Glyma15g11120 | -5.5 | 0.001 |
| Glyma02g37160 | -5.5 | 0.000 |
| Glyma02g36490 | -5.5 | 0.028 |
| Glyma06g13400 | -5.5 | 0.001 |
| Glyma13g36900 | -5.5 | 0.041 |
| Glyma02g31140 | -5.5 | 0.007 |
| Glyma05g28170 | -5.5 | 0.009 |
| Glyma02g43440 | -5.5 | 0.002 |
| Glyma01g03880 | -5.5 | 0.002 |
| Glyma08g39330 | -5.5 | 0.030 |
| Glyma05g21820 | -5.6 | 0.000 |
| Glyma18g45420 | -5.6 | 0.000 |
| Glyma01g40460 | -5.6 | 0.000 |
| Glyma07g06290 | -5.6 | 0.046 |
| Glyma12g33560 | -5.6 | 0.021 |
| Glyma20g26510 | -5.6 | 0.013 |
| Glyma11g35700 | -5.6 | 0.007 |
| Gm12:33333701 | -5.6 | 0.017 |
| Glyma07g29990 | -5.6 | 0.000 |
| Glyma04g01640 | -5.6 | 0.001 |
| Glyma08g41530 | -5.6 | 0.013 |
| Glyma16g24440 | -5.7 | 0.001 |
| Glyma09g40990 | -5.7 | 0.002 |
| Glyma09g41000 | -5.7 | 0.001 |
| Glyma19g42630 | -5.7 | 0.036 |
| Glyma05g38260 | -5.8 | 0.001 |
| Glyma12g30870 | -5.8 | 0.000 |
| Glyma01g39810 | -5.8 | 0.000 |
| Glyma09g03970 | -5.8 | 0.037 |
| Glyma11g12450 | -5.8 | 0.005 |
| Glyma17g05360 | -5.8 | 0.008 |
| Glyma04g04460 | -5.8 | 0.000 |
| Glyma05g33190 | -5.8 | 0.013 |
| Glyma07g01730 | -5.8 | 0.000 |
| Glyma03g28740 | -5.8 | 0.000 |
| Glyma12g30730 | -5.8 | 0.009 |
| Glyma02g39960 | -5.8 | 0.000 |
| Glyma14g05250 | -5.9 | 0.000 |
| Glyma03g28520 | -5.9 | 0.003 |
| Glyma16g27990 | -5.9 | 0.004 |
| Glyma01g06640 | -5.9 | 0.008 |
| Glyma09g08120 | -5.9 | 0.000 |
| Glyma17g01580 | -5.9 | 0.000 |
| Glyma19g02810 | -5.9 | 0.001 |
| Glyma06g05200 | -5.9 | 0.004 |
| Glyma19g25570 | -5.9 | 0.005 |
| Glyma08g12020 | -5.9 | 0.002 |
| Glyma06g04550 | -5.9 | 0.000 |
| Glyma19g43920 | -5.9 | 0.012 |
| Glyma18g14640 | -5.9 | 0.046 |
| Glyma02g07610 | -5.9 | 0.006 |
| Glyma14g39880 | -6.0 | 0.001 |
| Glyma15g35130 | -6.0 | 0.026 |
| Glyma01g06030 | -6.0 | 0.000 |
| Glyma14g14220 | -6.0 | 0.000 |
| Glyma14g28090 | -6.0 | 0.046 |
| Glyma13g26600 | -6.0 | 0.009 |
| Glyma09g02550 | -6.0 | 0.000 |
| Glyma02g03680 | -6.0 | 0.001 |
| Glyma08g47160 | -6.0 | 0.001 |
| Glyma06g43630 | -6.0 | 0.000 |
| Glyma01g37930 | -6.0 | 0.019 |
| Glyma19g02510 | -6.0 | 0.001 |
| Glyma09g31260 | -6.0 | 0.006 |
| Glyma12g00890 | -6.0 | 0.001 |
| Glyma13g36870 | -6.1 | 0.008 |
| Gm02:30379053 | -6.1 | 0.009 |
| Glyma10g00440 | -6.1 | 0.000 |
| Glyma08g01180 | -6.1 | 0.001 |
| Glyma12g32390 | -6.1 | 0.004 |
| Glyma12g35430 | -6.1 | 0.004 |
| Glyma10g39760 | -6.1 | 0.001 |
| Glyma04g00680 | -6.1 | 0.001 |
| Glyma09g30910 | -6.1 | 0.002 |
| Glyma17g10720 | -6.2 | 0.008 |
| Glyma16g00980 | -6.2 | 0.000 |
| Glyma01g01310 | -6.2 | 0.013 |
| Glyma02g00280 | -6.2 | 0.000 |
| Glyma06g20830 | -6.2 | 0.000 |
| Glyma18g49570 | -6.3 | 0.000 |
| Glyma05g14800 | -6.3 | 0.003 |
| Glyma20g05530 | -6.3 | 0.004 |
| Gm20:36291956 | -6.3 | 0.006 |
| Glyma14g03210 | -6.4 | 0.000 |
| Glyma13g41650 | -6.4 | 0.000 |
| Glyma10g39440 | -6.4 | 0.004 |
| Glyma11g10330 | -6.4 | 0.012 |
| Glyma07g08760 | -6.4 | 0.001 |
| Glyma13g24720 | -6.4 | 0.025 |
| Glyma09g28460 | -6.4 | 0.001 |
| Glyma15g00450 | -6.4 | 0.006 |
| Glyma13g19220 | -6.4 | 0.004 |
| Glyma16g26630 | -6.4 | 0.005 |
| Glyma14g15120 | -6.5 | 0.000 |
| Glyma17g10050 | -6.5 | 0.000 |
| Glyma18g15920 | -6.5 | 0.021 |
| Glyma13g27050 | -6.5 | 0.001 |
| Glyma06g43760 | -6.5 | 0.000 |
| Glyma15g01850 | -6.7 | 0.008 |
| Glyma14g37430 | -6.7 | 0.002 |
| Glyma11g07420 | -6.7 | 0.008 |
| Glyma06g01710 | -6.7 | 0.002 |
| Glyma19g26710 | -6.7 | 0.001 |
| Glyma10g32930 | -6.7 | 0.002 |
| Glyma13g39440 | -6.7 | 0.001 |
| Glyma05g37730 | -6.8 | 0.000 |
| Glyma08g42010 | -6.9 | 0.001 |
| Glyma01g35620 | -6.9 | 0.000 |
| Glyma12g32160 | -6.9 | 0.000 |
| Glyma08g36590 | -6.9 | 0.043 |
| Glyma08g12750 | -6.9 | 0.000 |
| Glyma08g01860 | -6.9 | 0.000 |
| Glyma08g25090 | -6.9 | 0.011 |
| Glyma19g35090 | -6.9 | 0.040 |
| Glyma18g35410 | -6.9 | 0.042 |
| Glyma15g30150 | -7.0 | 0.010 |
| Glyma04g04420 | -7.0 | 0.050 |
| Glyma04g33610 | -7.1 | 0.000 |
| Glyma07g39160 | -7.1 | 0.002 |
| Glyma08g39700 | -7.1 | 0.001 |
| Glyma12g33600 | -7.2 | 0.043 |
| Glyma12g29530 | -7.3 | 0.040 |
| Glyma02g43680 | -7.3 | 0.000 |
| Glyma13g30410 | -7.3 | 0.006 |
| Glyma06g08610 | -7.3 | 0.002 |
| Glyma08g18200 | -7.4 | 0.010 |
| Glyma01g04150 | -7.4 | 0.021 |
| Glyma20g35520 | -7.4 | 0.027 |
| Glyma13g43970 | -7.4 | 0.002 |
| Glyma16g05760 | -7.4 | 0.000 |
| Glyma14g05200 | -7.4 | 0.010 |
| Glyma05g30380 | -7.5 | 0.000 |
| Glyma09g37290 | -7.5 | 0.000 |
| Glyma01g04070 | -7.5 | 0.000 |
| Glyma18g43920 | -7.5 | 0.000 |
| Glyma16g04960 | -7.6 | 0.031 |
| Glyma08g47380 | -7.6 | 0.001 |
| Glyma13g38310 | -7.6 | 0.001 |
| Glyma08g31890 | -7.6 | 0.000 |
| Glyma08g21730 | -7.7 | 0.000 |
| Glyma07g02060 | -7.7 | 0.000 |
| Glyma13g21380 | -7.7 | 0.022 |
| Glyma07g10820 | -7.8 | 0.022 |
| Glyma08g22340 | -7.8 | 0.000 |
| Glyma08g16220 | -7.8 | 0.000 |
| Glyma07g16690 | -7.9 | 0.001 |
| Glyma17g32100 | -7.9 | 0.000 |
| Glyma15g13460 | -8.0 | 0.016 |
| Gm08:14527616 | -8.0 | 0.046 |
| Glyma01g45570 | -8.1 | 0.000 |
| Glyma04g09740 | -8.3 | 0.011 |
| Glyma05g23170 | -8.4 | 0.007 |
| Glyma05g29630 | -8.5 | 0.000 |
| Glyma06g01730 | -8.5 | 0.030 |
| Glyma11g15440 | -8.5 | 0.001 |
| Glyma11g10460 | -8.9 | 0.000 |
| Glyma07g05500 | -9.0 | 0.000 |
| Gm11:7469920 | -9.2 | 0.001 |
| Glyma17g37900 | -9.3 | 0.000 |
| Glyma13g22940 | -9.5 | 0.000 |
| Glyma14g33480 | -9.8 | 0.000 |
| Gm08:6264434 | -9.9 | 0.000 |
| Glyma11g25650 | -10.0 | 0.005 |
| Glyma19g06160 | -10.4 | 0.003 |
| Glyma19g43940 | -10.4 | 0.000 |
| Glyma04g35030 | -10.4 | 0.000 |
| Glyma03g41330 | -10.4 | 0.000 |
| Glyma11g02440 | -10.5 | 0.000 |
