# Supplementary material for: Comparisons of the Effects of Elevated Vapor Pressure Deficit on Gene Expression in Leaves among Two Fast-Wilting and a Slow-Wilting Soybean
Source: PLoS One. 2015 Oct 1;10(10):e0139134. doi: 10.1371/journal.pone.0139134 (PMC4591296; doi:10.1371/journal.pone.0139134)
Supplement: S4 Table — Table includes GO categories with FDR <0.05. (DOCX) [file pone.0139134.s007.docx]

**Supporting Table 4.** List of over represented GOs in PI 416937 from the up regulated genes list under high VPD. GO categories with FDR>0.05 were shown in the table.

| **GO term** | **Ontology** | **Description** | **Percentage of input list** | **Percentage of BG/Ref** | **FDR** |
| --- | --- | --- | --- | --- | --- |
| GO:0005576 | Biological | Extracellular region | 3.01 | 0.59 | 0.00 |
| GO:0048046 | Biological | Apoplast | 1.67 | 0.26 | 0.01 |
| GO:0055114 | Cellular Component | Oxidation reduction | 20.07 | 8.72 | 0.00 |
| GO:0009607 | Cellular Component | Response to biotic stimulus | 2.01 | 0.20 | 0.00 |
| GO:0016705 | Molecular Function | Oxidoreductase activity, acting on paired donors, with incorporation or reduction of molecular oxygen | 10.37 | 2.56 | 0.00 |
| GO:0020037 | Molecular Function | Heme binding | 9.03 | 2.16 | 0.00 |
| GO:0046906 | Molecular Function | Tetrapyrrole binding | 9.03 | 2.17 | 0.00 |
| GO:0005506 | Molecular Function | Iron ion binding | 9.70 | 2.54 | 0.00 |
| GO:0016491 | Molecular Function | Oxidoreductase activity | 22.07 | 10.25 | 0.00 |
| GO:0016758 | Molecular Function | Transferase activity, transferring hexosyl groups | 7.69 | 2.37 | 0.00 |
| GO:0009055 | Molecular Function | Electron carrier activity | 8.03 | 2.97 | 0.00 |
| GO:0016757 | Molecular Function | Transferase activity, transferring glycosyl groups | 7.69 | 2.98 | 0.00 |
| GO:0016746 | Molecular Function | Transferase activity, transferring acyl groups | 5.02 | 1.67 | 0.00 |
| GO:0016747 | Molecular Function | Transferase activity, transferring acyl groups other than amino-acyl groups | 4.68 | 1.51 | 0.00 |
| GO:0016706 | Molecular Function | Oxidoreductase activity, acting on paired donors | 3.34 | 0.89 | 0.01 |
| GO:0046914 | Molecular Function | Transition metal ion binding | 12.37 | 7.19 | 0.02 |
| GO:0003700 | Molecular Function | Transcription factor activity | 8.03 | 3.99 | 0.02 |
| GO:0046872 | Molecular Function | Metal ion binding | 15.72 | 9.84 | 0.02 |
| GO:0043169 | Molecular Function | Ccation binding | 15.72 | 9.87 | 0.02 |
| GO:0043167 | Molecular Function | Ion binding | 15.72 | 9.87 | 0.02 |
| GO:0016762 | Molecular Function | Xyloglucan:xyloglucosyl transferase activity | 1.67 | 0.26 | 0.02 |
| GO:0003854 | Molecular Function | 3-beta-hydroxy-delta5-steroid dehydrogenase activity | 2.34 | 0.61 | 0.03 |
| GO:0033764 | Molecular Function | Steroid dehydrogenase activity, acting on the CH-OH group of donors, NAD or NADP as acceptor | 2.34 | 0.61 | 0.03 |
| GO:0016229 | Molecular Function | Steroid dehydrogenase activity | 2.34 | 0.61 | 0.03 |
